# The adaptation property in non-equilibrium chemical systems

**DOI:** 10.1007/s00285-026-02344-y

**Published:** 2026-02-13

**Authors:** E. Franco, J. J. L. Velázquez

**Affiliations:** University of Bonn: Rheinische Friedrich-Wilhelms-Universitat Bonn, Bonn, Germany

**Keywords:** Non-equilibrium systems, Adaptation property, Detailed balance., 92B, 34A, 37N25, 92C40, 92B05

## Abstract

The goal of this paper is to understand the relationship between the property of adaptation (i.e. the insensitivity of the concentration of some substances on the change of the concentration of some chemical signals) and the absence of thermal equilibrium of the system. We prove that, unless the conserved quantities of a signalling system satisfy a very specific factorization assumption, adaptation cannot be achieved in a robust manner by a general class of systems that satisfy the detailed balance property and that do not exchange substances or energy with the environment. We also prove that robust adaptation can be achieved by systems that satisfy the detailed balance property, but exchange substances with the environment. We also recover classical adaptation mechanisms freezing the concentrations of some substances in systems with mass conservation and with detailed balance in some suitable limit regimes.

## Introduction

### Goal and motivation of the paper

Detailed balance is a property that every kinetic model that is not exchanging neither mass or free energy with the surroundings must satisfy at the microscopic level (see (Avanzini et al. 2024, 2025; Polettini and Esposito 2014)). This is a consequence of the fact that the laws describing the dynamics of the particles are invariant under time reversal (see for instance (Boltzmann 1964; Boyd 1974)). As a matter of fact, using thermodynamics arguments, it can be shown that the detailed balance property holds also for kinetic systems at constant temperature that do not exchange matter and Gibbs free energy with the environment. This does not mean that it is not possible to use models in which detailed balance fails in order to model kinetic systems. Indeed, the failure of detailed balance is a measure of the exchange of free energy and matter with the surroundings. Equivalently, the failure of detailed balance provides an estimate of the lack of equilibrium of the system. In most biological situations the absence of equilibrium is a consequence of the fact that the concentrations of some energetic molecules like ATP, ADP are at non equilibrium values (see for instance (Alberts et al. 2022)).

Many biochemical networks that are used to model biological systems do not satisfy the detailed balance property. As indicated above this is due to the fact that they are operating “out of equilibrium” and therefore must spend energy, usually in the form of ATP, in order to function. Typical examples of biochemical systems that need to be modelled by means of system of equations for which the detailed balance property fails are the sensing models of chemotaxis (see (Alon 2019; Milo and Phillips 2015; Phillips et al. 2012)) or the kinetic proofreading models that describe the mechanisms that correct errors in processes like transduction, transcription or antigen recognition by the immune system. In all these processes the kinetic proofreading mechanism need to distinguish between different molecules (see (Alon 2019; Franco and Velázquez 2025b; Hopfield 1974; Phillips et al. 2012))

Therefore, it is natural to ask which functions of a biological system require lack of equilibrium (hence either the system exchanges chemicals or free energy with the environment) and which ones can be performed in equilibrium conditions (i.e. in closed systems that satisfy detailed balance and are conservative). As indicated above, this is equivalent to understand which biological functions require an active use of energy and/or fluxes of substances from/into the environment and which biological functions, instead, can take place in a passive manner, in equilibrium conditions. Although a large number of biological processes require the use of energetic molecules, some processes take place without any need to use ATP or any other molecule. For instance in the DNA replication process, a particular class of enzymes, called *topoisomerases I*, catalyze changes in the DNA topology that allow to relax any tension in the DNA helix. It seems that this process does not require any energy supply (see [Alberts et al. (2022), Chapter 5]).

In this paper we will focus on a property that is common for many signalling systems, namely the so called *adaptation* property. This consists in the fact that many signalling systems react to the changes in a signal (in time or in space) more than to the absolute values of the signal concentrations. In other words, many biological systems respond to a stimulus, but after a transient time they return to the basal activity level that they had before the stimulus. A biological situation in which the adaptation property arises is in the signal processing of bacterial chemotaxis. The earliest models for these processes exhibiting adaptation were obtained in the classical paper by Barkai and Leibler (see (Barkai and Leibler 1997)). The adaptation property of the response to the signal yielding chemotaxis in eukaryotes cells was experimentally established in Parent and Devreotes (1999). As a rule, the adaptation property is ubiquitous in sensory systems, for instance in visual sensory systems (see (Alon 2019; Segel et al. 1986)). The biological role of adaptation in these systems is that it allows to obtain responses under a large variety of external backgrounds, including different levels of light intensity, or chemical concentrations. Another example of a biological process in which adaptation plays a role is the so called mitogen signalling, i.e. the mechanism that induces or enhances cell division. In this particular case the adaptation property plays a crucial role in order to avoid uncontrolled cell proliferation (cf. Ferrell (2016)).

The main purpose of this paper is to understand if a chemical network that exhibits robust adaptation can function in equilibrium conditions or if, on the contrary, the adaptation property requires an active process that is out of equilibrium. Since there exist biological processes that take place without any external input of energy and without any external input of substances, i.e. at equilibrium conditions, understanding if adaptation requires non equilibrium conditions is a relevant question.

### Signalling systems

In this paper we study the relation between detailed balance and the adaptation property described above. To this end, we define a class of ideal *signalling systems*. These are kinetic systems in which the concentration of one substance, that we call *signal*, changes in time due to influxes and outfluxes of signal. Therefore, the change of signal concentration does not follow the dynamics imposed by the reactions in the kinetic system, instead its evolution is prescribed by some external boundary condition.

The models that we study in this paper are variations of the classical kinetic systems. These are composed by a set of substances $\Omega =\{ 1, \dots , N \}$, a set of chemical reactions and chemical rates. The dynamics of the vector of concentrations $n =(n_1, \dots , n_N)^T\in \mathbb {R}^N$ is given by

1.1 $$\begin{aligned} \frac{d n_i}{ dt} (t)= J_i(n), \quad i \in \{ 1, \dots , N \}. \end{aligned}$$

Here $$J_i $$ represent the net flux, of chemicals at state $$i \in \Omega $$ due to the chemical reactions taking place in the network, their precise form will be given later. In order to have systems that are consistent with thermodynamics we must assume that the detailed balance property holds and that the system is conservative. This means that we can assign to each substance a positive number *m*. The simplest choice is to take *m* as the number of atoms of a molecule. Under these assumptions the equation (1.1) describes the dynamics of an isolated isothermal chemical system that converges to a steady state as time tends to infinity.

More interesting dynamics can be obtained when there are fluxes of chemicals between the environment and the kinetic system. In this case the dynamics of the vector of concentrations is given by

1.2 $$\begin{aligned} \frac{d n_i}{ dt} (t)= J'_i(n) + J_i^{ext} (t), \quad i \in \{ 1, \dots , N' \} \end{aligned}$$

where $J_i^{ext}(n)$ is the net flux of substance *i* form the environment. If the chemical reactions associated with the fluxes $J_i$ are such that the corresponding kinetic system (1.1) satisfies the detailed balance property and it is conservative we say that the system is *thermodynamically admissible* (see (Avanzini et al. 2024, 2025; Polettini and Esposito 2014)). We recall here a result that has been proven in Franco and Velázquez (2025a) and that we refer to as completion theorem. This theorem states that for any bidirectional kinetic system modeled by (1.1) it is possible to find an extended system, with a larger number of substances (i.e. $N'>N$), that satisfies detailed balance, is conservative (i.e. is thermodynamically admissible), it can be written as (1.2) if we assume that the concentrations of substances $i \in \{ N+1, \dots , N' \}$ are frozen to constant values, and in addition it reduces to (1.1) for the evolution of the substances $i \in \{ 1, \dots , N \}$. In this way every signalling system can be extended to a completed thermodynamically admissible system.

In order to justify the previous definition of thermodynamically admissible systems we just notice that as mentioned above, the detailed balance is a property that, at the fundamental level, must be satisfied by any chemical network at constant temperature that do not exchange chemicals with the environment (see for instance (Avanzini et al. 2024, 2025; Franco and Velázquez 2025a; Polettini and Esposito 2014)). Nevertheless a chemical system exchanging substances with the environment can be effectively described by a system of equations for which detailed balance does not hold. In other words, open systems exchanging matter with the environment, or that are in contact with reservoirs (or *chemostats*), can be effectively described by systems for which detailed balance fails (see (Polettini and Esposito 2014; Yang et al. 2021)). In Franco and Velázquez (2025a) we have obtained some rigorous mathematical justification of this fact. We proved there that kinetic systems for which detailed balance fails can be obtained starting from kinetic systems for which detailed balance holds, freezing the values of some of the concentrations. To freeze these concentrations requires to introduce some additional terms in the equations. These terms describe fluxes of the frozen substances to or from the system. The presence of these fluxes indicates that we are dealing with an open system exchanging matter and free energy with the surroundings. In particular if the fluxes of free energies and matter are different from zero at steady state, it turns out that the concentrations of the substances are out of equilibrium, i.e. they are not described by means of Gibbs distributions. The precise conditions on the concentration of the frozen substances, as well as in the network topology, that are required to obtain non trivial fluxes have been studied in detail in Franco and Velázquez (2025a).

In this paper we are mostly concerned about a particular type of systems of the form (1.2) that we call signalling systems. We will denote the substances in the system as $\Omega =\{1, 2, \dots , N \}$ where (1) refers to a signal and $\{ 2, \dots , N\}$ are the substances in the network, possibly including the receptors that detect the signal. We will assume that the concentration of signal $n_1$ is a given function *f*(*t*). Hence the ODEs describing the change in time of the vector of the concentration $n =(n_1, n_2, \dots , n_N)^T$ in the signalling system can be equivalently formulated as

1.3 $$\begin{aligned} \frac{d n_i}{ dt} (t)&= J_i(n) + J_i^{ext}(n), \quad i \in \{2, \dots , N \} \nonumber \\ n_1(t)&=f(t). \end{aligned}$$

The definition of signalling systems considered in this paper has analogies with the definition of signalling system studied in Shinar and Feinberg (2010); Sontag (2010).

In this paper we say that a signalling system of ODEs of the form (1.3) for the evolution of the concentrations $$(n_1, n_2, \dots , n_N )^T $$ is thermodynamically admissible if the system of reactions encoded in the fluxes $$J_i $$ for $$i \in \Omega $$ satisfies the detailed balance property and is conservative. A thermodynamically admissible signalling system is *closed* if $$J^{ext}_i (t)=0$$ for every $$i \in \{2, \dots , N \} $$ and open if there exists at least a substance $$i \in \{2, \dots , N\} $$ such that $$J_{i}^{ext}(t) \ne 0 $$ for some range of times.

In this work we will assume that the external fluxes $J^{ext}_i$ are prescribed in such a way that $n_i (t)=c_i$ for every $i \in \{ k +1, \dots , N\}$, for some $k \ge 1$, and that the rest of the concentrations $n_i (t)$, $i \in \{ 2, \dots , k\}$, evolve according to the chemical reactions modeled by means of $J_i(n)$. Therefore these concentrations are not affected by external fluxes. Then the fluxes $J^{ext}_i$ are such that $J^{ext}_i(t) = -J_i(n(t))$ for $i \in \{ k +1, \dots , N\}$ and $J_i^{ext} =0$ for $i \in \{ 2, \dots , k \}$.

We will assume that the concentration of signal *f*(*t*) tends to a constant value as the time *t* tends to infinity. It is reasonable to expect that changes in the concentration of the signal yield changes in some of the concentrations of other substances in the signalling system. In particular, it is relevant to understand how the concentration(s) of a substance (or group of substances) changes in time after modifying the concentration of the signal $n_1$.

The main goal of the paper is to understand under which conditions systems of the form (1.3) with the properties stated above satisfy the property of adaptation. In particular, the aim is to study how the concentration of a specific substance changes in time after changes in the signal concentration. We refer to this substance (or substances) as the *product* (*p*) of the signalling system and the change in the concentration of product is the *response* of the signalling system to the changes in the signal concentration.

In typical signalling situations the function *f* begins from a given constant value and converges to another constant values for large times. In this paper we study signalling systems in which the signal converges to a steady state sufficiently fast (see Assumption 2.14 for the precise assumptions on the speed of convergence). These models of signalling systems mimic the structure of biological signalling systems. One example of simple signalling system is the two-component signalling pathway. This pathway consists of two steps, as a first step a ligand binds to a cell receptor. The complex ligand receptor then undergoes a sequence of chemical reactions, that usually requires consumption of molecules (for instance ATP), and finally forms a product (we refer to Milo and Phillips (2015); Phillips et al. (2012) for more details on these models).

We assume that the signalling systems that we study are endowed with mass action kinetics and the results that we prove in the paper are valid in principle only for these systems. The mass action assumption is a natural choice of kinetics for kinetic systems mainly driven by binary reactions. It is well known that other types of kinetics, as for instance the Michaelis-Menten kinetics, can be derived as limit of systems endowed with mass action kinetics (see (Goldbeter and Koshland 1981; Segel and Slemrod 1989)). Most of the results that we prove in this paper for general systems are valid only for kinetic systems described by the mass action law and with chemical rates that are of order one. A question that would be relevant to understand is to determine if the adaptation property can be inherited by systems that are obtained as a limit of bidirectional networks with mass action that are thermodynamically consistent. Some particular examples of these limit regimes will be discussed in Section 4.

### The property of adaptation in thermodynamically admissible systems

As indicated above, our goal introducing signalling systems is to study the adaptation property for these types of systems. More precisely, we say that a signalling system satisfies the adaptation property if

- the concentration of product (*p*) must change in a non trivial manner after changes of the concentration of signal.
- after a transient time the concentration of product (*p*) returns to the pre-signal levels.

**Fig. 1:**
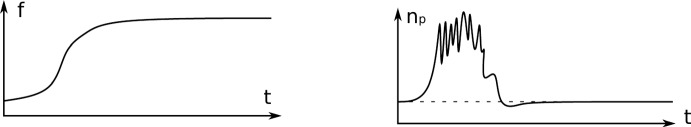
On the left we plot the function *f*, describing the change in time of the concentration of signal. The function *f* tends to a constant value as $t\rightarrow \infty$. On the right we plot $n_p(t)$, which is the concentration of product (*p*), for a signalling system that satisfies the adaptation property. Notice that the concentration of product changes as the signal changes. However it returns to the pre-signal values as time tends to infinity

See Figure 1 for a graphical representation of the adaptation property. Most of the studies of adaptation in the literature focus in a condition that is similar to point (b) above, or specifically in the independence of the values of the concentration of some substances at steady state on the chemical rates. In this paper we will consider also the non trivial response of the signal (point (a) above) because as we will see in Section 3.1 there are examples of connected (cf. Definition 3.1) signalling systems in which there is no response to any signal.

In order to study the property of adaptation it is convenient to study the conservation laws associated with the system (1.1). A conservation law is a linear combination of the concentrations that remains constant in time. The space of conservation laws is a linear subspace of $\mathbb {R}^{N}$.

In this paper we restrict our attention to thermodynamically admissible systems. For this class of systems we prove the following results.

1. Closed signalling systems cannot have robust adaptation, unless a factorization property involving the substances in $\Omega$ and the conservation laws holds.
2. There exists a class of open systems for which the property of robust adaptation holds.
3. We obtain specific signalling systems that are thermodynamically admissible which converge, in suitable asymptotic regimes, to classical models that have the property of adaptation.

We now comment these results.

We begin discussing point 1. We recall that closed signalling systems have been defined by means of the system of equations (1.3) with $J_i^{ext} =0$, i.e. the equations modeling them are

1.4 $$\begin{aligned} \frac{d n_i}{ dt} (t)&= J_i(n) , \quad i \in \{2, \dots , N \} \nonumber \\ n_1(t)&=f(t). \end{aligned}$$

We notice that the equation for the signal $$n_1 $$ can be rewritten as $$\frac{d n_1}{ dt } = J_1(n) + J_1^{ext}(t) =0$$ where $$J_1^{ext}$$ must be selected in a particular way in order to have $$n_1(t)=f(t)$$. Therefore a closed signalling system can be interpreted as a thermodynamically admissible system for which the only substance exchanged with the environment is the signal. The factorization property for closed thermodynamically admissible signalling systems means that there exists a basis of conservation laws $$\{ m_\ell \}_{\ell = 1}^L $$, $$m_\ell \in \mathbb {R}_+^N $$ such that

1.5 $$\begin{aligned} M = \begin{bmatrix} m_1^T \\ m_2^T \\ \dots \\ m_L^T \end{bmatrix} = \begin{bmatrix} A & 0 \\ 0 & B \end{bmatrix} \end{aligned}$$

with $A \ne 0$ and $B \ne 0$. In this representation each column is associated to one of the reactants. Then the factorization property requires in addition that the signal must be associated with one of the columns intersecting *A* and the product(s) is(are) associated with one (or several) of the columns intersecting *B*. This means that if we think about the conservation laws as the components of the molecules of the system the factorization property implies that the signal and the products consist of completely different ingredients.

We now discuss the point 2. above. The class of open systems referred in this point is a class of systems of the form (1.3) that has the property that removing the equations that contain non trivial external fluxes we obtain a system that satisfies detailed balance but that is not conservative. Moreover, for this reduced system all the conservation laws $\{ m_\ell \}_{\ell = 1 }^L$ must vanish in the product/s.

It is important to notice that the concept of robustness that we use in this paper is different from the one that is commonly used in the literature studying adaptation. In particular, we only allow for perturbations of the chemical rates that do not break the property of detailed balance in the thermodynamically admissible system (1.3). In the previous studies of robust adaptation this restriction is not assumed in the perturbation of the chemical rates. A similar notion of perturbation has been used in Franco et al. (2024) as well as a description of the corresponding topology on the networks. Notice however that the scope of the paper (Franco et al. 2024) is different to the one in this paper. The question that has been addressed in Franco et al. (2024) is to characterize the chemical systems that satisfy the detailed balance property in terms of measurements of the evolution in time of some concentrations. This problem can be thought as an inverse problem.

We comment on point 3. Our definition of thermodynamically admissible requires the chemical reactions to be bidirectional. It is well known that systems with the detailed balance condition can be considered effectively as one-directional if the energy of the products is much smaller than the energy of the reactants (Gorban and Yablonsky 2011). As a matter of fact one-directional networks have been extensively used in the modeling of biochemical pathways, as well as in the mathematical literature of kinetic systems. Actually the mathematical conditions required to show that a chemical network containing one or several one-directional reactions can be obtained as a limit of a system with detailed balance (and therefore bidirectional) has been studied in detail in Gorban and Yablonsky (2011). It turns out that most of the models of adaptation used in the literature contain at least one reaction that is one-directional. In Section 4 we explain how these one-directional reactions can be obtained as limits of thermodynamically admissible signalling systems of the form (1.3) for some of the classical models of adaptation.

One of the issues that we discuss is one of the earliest models of adaptation studied by Segel at el in Segel et al. (1986). Further studies of this model have been made in Walz and Caplan (1987). An interesting feature of this model is that, in our terminology, it is an example of closed signalling system that satisfies the property of adaptation. A priori this seems to contradict the point 1. above. However it turns out that this is not the case because the property of adaptation in the model in Segel et al. (1986) is not robust. We will explain this in detail in Subsection 3.5.

The definition of adaptation that we use in this paper, rigorously stated in Definition 2.13, is a bit more restrictive than the definition of adaptation given in Hirono et al. (2025). Indeed in this paper we require, in addition to the invariance of the steady state concentrations under changes of the signal, to have a non-trivial response for non constant signals. We state the main results in an informal way in order to avoid most of the mathematical notation. The point 1 mentioned above is contained in the following theorem.

#### Theorem 1.1

(Stable adaptation is impossible in closed systems unless a factorization assumption holds) Assume that a signalling system is closed (i.e. it is thermodynamically admissible and the only non trivial fluxes are due to the signal). Then the signalling system does not satisfy the adaptation property unless the reaction rates are fine tuned or the conserved quantities satisfy a factorization assumption.

The rigorous statement of the above result is Theorem 3.12. The factorization assumption mentioned (cf. (1.5)) in the theorem above, will be defined later in Section 3.

The onset of a non trivial response to an external signal for the signal models described in the point 2 above is the content of the following result.

#### Theorem 1.2

(Response in a connected system) Consider a connected signalling kinetic system satisfying the detailed balance property (but not necessarily conservative). Then changes in the signal concentration produce changes in the concentrations of all the substances in the system, unless the parameters are fine tuned.

The proof of Theorem 1.2 for a general class of networks will reduce to study in detail the connectivity (see Definition 3.1) of the set of different substances. More precisely we can introduce an equivalence relation between two substances of a system stating that they are in the same equivalence class if there exists a reaction to which both of them belong. For systems with detailed balance we prove that the connectivity property implies that the property (a) in the definition of adaptation holds in a robust manner.

The main ingredient in order to prove the point 2. above is to notice that if a connected signalling system satisfies the detailed balance property (i.e. is thermodynamically admissible) and there are non trivial fluxes of one or more substances different from the signal, then the property (b) in the definition of adaptation also holds in a robust manner. This is the main content of Proposition 3.5 that we reformulate in an informal manner here.

#### Theorem 1.3

(Adaptation in kinetic systems with the detailed balance property) Assume that the system (1.4) is connected, it satisfies the detailed balance property and is not conservative (i.e. it exchanges at least one substance with the environment). Then, unless the parameters are fine tuned, the adaptation property holds for at least one of the substances in the system.

The theorem above indicates how to construct signalling systems that have the adaptation property and that are bidirectional. Indeed, it is enough to construct a kinetic system in such a way that one substance is not conserved in the network and then to select the chemical rates in such a way that detailed balance holds. As far as we know these class of signalling systems that satisfy the adaptation property had not been described before.

### Comparison with some previous results on the property of adaptation

The adaptation property, as well as the closely related notion of homeostasis, have been extensively studied by researchers working in systems biology, mathematical biology and biophysics. The earliest studies were concerned with understanding specific systems, for instance the methylation modifications yielding chemotaxis in *E. coli.* In particular, some specific models, well suited to specific biological situations were introduced. One of the earliest examples is the classical Barkai-Leibler model, which is a mechanism leading to robust adaptation in simple signal transduction networks. These mechanisms applies in particular to bacterial chemotaxis. Since adaptation and homeostasis are ubiquitous in biology, a large number of specific biological problems have been mathematically modeled by means of systems of ODEs (some examples are calcium homeostasis (El-Samad et al. 2002), visual sensory systems (Segel et al. 1986; Walz and Caplan 1987), yeast osmoregulation (Muzzey et al. 2009)).

On the other hand, there have been several attempts to characterize the property of adaptation in terms of the mathematical structure of the kinetic systems. One of the directions that has been extensively studied is the characterization of the motives that yield the property of robust perfect adaptation (RPA). This has been made in Alon (2019); Ferrell (2016). Here the term perfect indicates that, after a transient time, the system returns exactly to the activity level that it had before the signal started. The term robust refers to the fact that the property of adaptation is stable upon perturbations of the chemical rates. It was noticed, by means of an exhaustive computational analysis of a large class of kinetic systems, that the adaptation property is associated to the presence of two specific motives, namely the incoherent feedforward (IFF) loops and the negative feedback (NFB) networks (cf. Ma et al. (2009)). Rigorous mathematical results in this direction have been obtained recently in Araujo and Liotta (2018); Reed et al. (2017); Wang et al. (2021).

It has also been noticed by several authors the close relation between the adaptation property and control theory (Alon 2019; Sontag 2010, 2013). Specifically, ideas of control theory have been used to explain the lack of sensitivity of the concentrations of some chemicals to the changes in some external concentrations (Aoki et al. 2019; Frei et al. 2022). In particular, the strong relation between the so called Integral feedback controller and the adaptation property, has been noticed by several authors (Alon 2019; Aoki et al. 2019; Frei et al. 2022).

Another approach that has been used to characterize the kinetic systems having the (RPA) property has been to derive suitable topological properties of the network which ensure it. One of the earliest papers in this direction is Shinar and Feinberg (2010). In this paper a sufficient topological condition for adaptation (i.e. independence of the steady state concentration of some product substance on the concentration of a signal) was found. A closely related direction that has been also extensively studied are the conditions required to have * buffering structures*. These are subnetworks with the property that the chemical rates of the reactions taking place in such subnetworks do not influence the steady concentrations outside them. A detailed characterization of the buffering structures has been obtained in Hirono et al. (2025); Hirono et al. (2023); Okada and Mochizuki (2016); Yamauchi et al. (2024). The sensitivity of the steady concentrations of some classes of networks in terms of the topological properties of the reactions have been considered also in Fiedler and Mochizuki (2015); Mochizuki and Fiedler (2015); Vassena and Matano (2017).

We now clarify an important notational issue. There is not a unified terminology concerning the term adaptation. The notion of adaptation studied in Hirono et al. (2025); Khammash (2021); Okada and Mochizuki (2016); Yamauchi et al. (2024) is different from the notion of adaptation that we use in this paper. Indeed, in our paper we study if the concentration of a product at steady state changes as the concentration of signal changes. Instead in Hirono et al. (2025); Khammash (2021); Okada and Mochizuki (2016); Yamauchi et al. (2024) the dependence on specific chemical rates of one or several concentrations at steady state is analysed. These two definitions of adaptation are different. However, we can relate the two properties as follows. It might happen that some of the chemical rates of an effective model are functions on the concentration of an external signal, which is not included explicitly in the model, but should be included if a more detailed modeling of the system is attempted. More precisely, assume that in the network there is a reaction with the form $(1)+(2)\rightarrow (3)$ taking place at a certain rate *K*. Assume that the concentration of the signal (1) is constant in time, then we can ignore the presence of the substance (1) in our model, and to replace the dependence on its concentration $n_{1}$ by means of the chemical rate of the effective reaction $(2)\rightarrow (3)$ which should be taken as $Kn_{1}$ . In these situations, the independence of one or several concentrations $\left\{ n_{\alpha }\right\} _{\alpha \in I},$ where *I* is a suitable set of indexes, on this effective chemical rate, would be equivalent to the independence of the concentrations $\left\{ n_{\alpha }\right\} _{\alpha \in I}$ on the signal $n_{1}.$ As a matter of fact, the situation becomes more complicated if the structure of conserved quantities is taken into account because changes in the concentrations of $n_1$ could have an effect on the concentration of the product. This issue will be discussed in more detail in Section 3.4.

In this paper we will use the term adaptation to describe the independence at steady state of the concentration of a substance (referred as product) in the concentration of another one (denoted as signal). We use the term homeostasis (=sensitivity) to refer to the independence of the steady state concentration of a set of products on the chemical rates. Actually, although the most common usage in the literature is to refer to adaptation as a property of the steady states, in this paper we will require also a non-trivial response in the concentration of the product in order to have adaptation. This makes sense, since we are interested in the analysis of signalling systems. We will provide an example of absence of response to the change of a signal in Section 3.1 (Example 3.2).

It is worth to distinguish between *perfect adaptation* and * asymptotic adaptation*. In the common usage in the literature, as well as in most of the paper, perfect adaptation refers to the invariance of one or several concentrations under changes of one or several signal concentrations (or chemical rates as in Hirono et al. (2023)). On the other hand, one can imagine biological situations in which the adaptation property takes place in practice due to the smallness (or largeness) of some of the chemical rates (or concentrations of substances). We will refer to this situation as asymptotic adaptation. We will show in Section 4 some situations for which there is perfect adaptation for an effective model which describes in an approximate way the dynamics of a model for which detailed balance holds and where there are external fluxes, under the assumption that some of the chemical rates tend to zero and some of the concentrations of chemicals are very large. Therefore, for this model, that in our notation would be thermodynamically admissible, asymptotic adaptation holds.

The main novelty of this paper, in comparison with the approach of the previous literature, is that we include explicitly in our analysis the assumption that, at the fundamental level, the only thermodynamically admissible models are those for which the detailed balance condition holds and they are conservative. This does not mean that in this paper we only consider models within this class (detailed balance and conservative). We also study in detail, effective models, that are obtained freezing some of the concentrations at some constant values. This is one of the most common ways of modelling systems out of equilibrium (cf. Avanzini et al. (2024, 2025); Franco and Velázquez (2025a)). To freeze the concentrations is also referred in the physical literature as to introduce a system of chemostats. The advantage of this approach is that it allows to quantify the fluxes of external substances required to obtain the lack of thermal equilibrium necessary to have the adaptation property. (See for instance Section 3.2). In typical biological systems, this could correspond to the amount of *ATP* transformed in *ADP* in the chemical reactions taking place in the system. .

We finally mention that an issue that has been extensively studied is to find properties more general than detailed balance that ensure convergence of the solution to a unique steady state. Two of the main properties of kinetic systems are complex balance, as well as topological properties that are measured for instance by the deficiency index (see for instance (Cappelletti and Wiuf 2016; Desvillettes et al. 2017; Feinberg 1972; Jia et al. 2021)). Another issue that have been much studied are the conditions that ensure the existence of multiple steady states for non conservative systems (see for instance (Craciun and Feinberg 2005, 2006, 2010)). Sufficient algebraic conditions for periodic oscillations in mass action systems has also been studied in great detail (see for instance (Blokhuis et al. 2025; Fiedler 1985; Vassena 2025)).

### Notation

In this paper we use the following notation. We define $$\mathbb {R}_+$$ and $$\mathbb {R}_*$$ to be given respectively by $$ \mathbb {R}_* = [0, \infty )$$ and $$ \mathbb {R}_+ = (0, \infty )$$. Moreover we denote with $$e_i \subset \mathbb {R}^n $$, for $$ i \in 1, \dots n $$, the vectors of the canonical basis of $$\mathbb {R}^n$$. Given two vectors $$v_1, v_2 \in \mathbb {R}^n $$ we denote with $$\langle v_1, v_2 \rangle $$ their euclidean scalar product in $$\mathbb {R}^n$$ and with $$v_1 \otimes v_2 $$ we denote their tensor product. Finally, given a vector $$v \in \mathbb {R}^n $$, we will denote with $$\exp (v)$$ the vector $$ (e^{v(i)})_{i=1}^n \in \mathbb {R}^n$$. If there is no risk of confusion we use the shorter notation $$e^v $$ to indicate $$\exp (v)$$. Similarly it will be useful to denote with $$\log (v) $$ the vector $$ (\log (v(i)))_{i=1}^n \in \mathbb {R}^n$$. Given a $$z \in \mathbb {R}^n$$, we denote with $$B_r (z)$$ the open ball of radius *r* around *z*, i.e.

$$ B_r(z)=\{ y \in \mathbb {R}^n : \Vert z-y \Vert < r \} $$

where $\Vert \cdot \Vert$ is the Euclidean norm.

Let $\mathcal {G} =(V, E)$ be a graph. A walk w in $\mathcal {G}$ is a finite non-null sequence $v_0 e_1 v_1 e_2 v_2 \dots e_k v_k$ whose terms are alternatively vertices and edges such that, $e_i=(v_{i-1}, v_i)$ for every $i \in \{ 1 \dots k \}$. Moreover, $\ell (w):= k$. A path is a walk in which all the edges and all the vertices are distinct. In Section 3.1 we will use often the notation $i \in w$ where *w* is a walk and $i \in V$ to indicate that the walk *w* contains the vertex *i*. Moreover, unless not otherwise specified, we say that $f \sim g$ as $t \rightarrow \infty$ (or as $t \rightarrow 0$) if $\lim _{t\rightarrow \infty } \frac{f(t) }{g(t) } =1$ (or $\lim _{t \rightarrow 0} \frac{f(t) }{g(t)}=1$).

**Plan of the paper**

The plan for this paper is the following. In Section 2 we introduce the class of signalling systems considered in this paper. To this end we briefly recall the definition of kinetic system (see Section 2.1) and the definition of detailed balance for kinetic models that we study (see Section 2.2). As a last step we introduce the definition of a signalling system as a kinetic system in which the signal changes in time according to a given function of time *f*. This is done in Section 2.3. Moreover, in this section we also prove that if the kinetic system underlying the signalling system satisfies the detailed balance property and if the function *f*, that prescribes the way in which the signal changes in time, converges sufficiently fast to a constant value, then the the vector of the concentrations of the substances in the system converges to a steady state as time tends to infinity.

In Section 3 we state and prove Theorem 1.2. As we will explain later, in order to prove Theorem 1.2 we assume that the kinetic system, underlying the signalling system under study, satisfies detailed balance property. The reason why we make this assumption is that kinetic systems with the detailed balance property have a structure that allows to prove that changes in the signal produce changes in the product. It would be an interesting problem to study this question in full generality, i.e. removing the detailed balance property. In Section 3.2 we introduce a mechanism yielding the adaptation property in a signalling system that satisfies the detailed balance property. In other words, we prove Theorem 1.3. In Section 3.3 we state one of the main theorems of the paper, i.e. we prove that, unless the factorization assumption on the conserved quantities holds, robust adaptation is impossible in closed systems. This is the statement of Theorem 1.1. In Subsection 3.4 we discuss some examples of signalling systems that do not study the property of detailed balance and that have the property of adaptation. In particular we analyse the relation between our results and the results in Hirono et al. (2025); Yamauchi et al. (2024). In Section 3.5 we discuss the results obtained in Segel et al. (1986); Walz and Caplan (1987) and their relation with the main theorems of this paper.

In Section 4 we review some models of adaptation that can be found in the literature and we examine them from the point of view of thermodynamic admissibility. The models we discuss include a linear version of the Barkai-Leibler model of bacterial chemotaxis, the classical model of adaptation proposed in Segel et al. (1986) and some of the models considered in Ferrell (2016). These models contain one-directional reaction and are not necessarily endowed with mass action kinetics. However we will show that they can be obtained starting from bidirectional mass action systems that satisfy the detailed balance property.

## Basic properties of signalling systems and the property of adaptation

The goal of this section is to give the definition of signalling system and collect some definitions that will be used later. As mentioned in the introduction a signalling system is a kinetic system where one substance, the signal, changes in time according to a given external boundary condition *f*. Therefore we start this section defining kinetic systems.

### Kinetic systems and their conservation laws

In this section we define the class of kinetic systems under consideration. A kinetic system is a set of of substances, a set of reactions and a set of reaction rate functions.

#### Definition 2.1

(*Kinetic system*) Let $\Omega :=\{ 1, \dots , N \}$. Let $r \ge 1$ and $\mathcal {R} := \{ R_1, \dots , R_r \}$ where $R_j \in \mathbb {Z}^N\setminus \{ 0\}$ for every $j \in \{ 1, \dots , r \}$. Let $\mathcal {K}: \mathcal {R} \rightarrow \mathbb {R}_+$ be a function. Then $(\Omega , \mathcal {R}, \mathcal {K})$ is a kinetic system.

The set $\Omega$ is the set of the substances in the system, $\mathcal {R}$ is the set of chemical reactions taking place in the network and $\mathcal {K} (R)$ is the rate of the reaction $R \in \mathcal {R}$. The function $\mathcal {K}$ is usually called reaction rate function. Later, to simplify the notation, we will indicate with $K_R$ the rate of the reaction $R \in \mathcal {R}$, i.e. $K_R:=\mathcal {K}(R)$.

Let $R \in \mathcal {R}$ be a reaction. We will use the following notation

$$ I(R):=\{ i \in \Omega : R(i) <0 \}, \quad F(R):= \{ i \in \Omega : R(i) >0 \} \ \text { and } \ D(R):=I(R) \cup F(R). $$

The set *I*(*R*) is the set of the initial substances of the reaction $R \in \mathcal {R}$ and the set *F*(*R*) is the set of the final substances of the reaction $R \in \mathcal {R}$. The set *D*(*R*) is the domain of the reaction, i.e. the set of the substances that take part to the reaction. In this paper we assume that $I(R) \cap F(R) = \emptyset$ for every $R \in \mathcal {R}$. This means that reactions of the form $(1) +(2) \rightarrow (2) +(3)$ as well as autocatalytic reactions are not considered in this paper. These type of reactions could be obtained as a limit of a chain of reactions of the class considered in Definition 2.1 with mass action (assuming that some of the chemical rates are large or small). Similarly kinetic systems that do not satisfy the mass action law can be obtained starting from mass action kinetic systems. A classical example is the derivation of the Michaelis-Menten law starting from bidirectional reactions with mass action kinetics (see (Segel and Slemrod 1989)).

Moreover, we assume that every substance in the system takes part in at least one reaction, this means for every $i \in \Omega$ there exists a $R \in \mathcal {R}$ such that $i \in D(R)$ and that $D(R)\ne \emptyset$ for every $R \in \mathcal {R}$.

We give the definition of conservation law.

#### Definition 2.2

(*Set of conservation laws*) The set $\mathcal {M}$ of conservation laws of a chemical network $(\Omega , \mathcal {R})$ is defined as

2.1 $$\begin{aligned} \mathcal {M} := {\operatorname {span}\{ R: R \in \mathcal {R} \}}^{\perp }. \end{aligned}$$

Let $$m \in \mathcal {M} $$, then

$$ m^T R = {\textbf {0}},\quad \forall R \in \mathcal {R}. $$

This is the reason why we refer to $$\mathcal {M} $$ as the set of the conservation laws. If $$n_0 \in \mathbb {R}^N $$ is the initial vector of concentrations $$n_0$$ and *n*(*t*) is the vector of concentrations at time $$t>0 $$, then

$$ m^T n_0 = m^T n(t) \quad \text { for every } t >0 \text { and for any } m \in \mathcal {M}. $$

We define now the set of physically relevant non-negative conservation laws $\mathcal {M}_+:=\mathcal {M} \cap \mathbb {R}_*^N.$

#### Definition 2.3

(*Conservative system*) We say that the kinetic system $(\Omega , \mathcal {R}, \mathcal {K} )$ is conservative if

2.2 $$\begin{aligned} \mathcal {M}_+ \cap \mathbb {R}_+^N \ne \emptyset . \end{aligned}$$

Notice that this is the same definition of conservative network given in Feinberg (2019).

It is possible to prove that it is always possible to find a positive basis of $\mathcal {M}$ when the kinetic system $(\Omega , \mathcal {R}, \mathcal {K} )$ is conservative. In particular, if the system is conservative then the cone $\mathcal {M}_+$ is a polyhedral cone whose extreme rays (we recall that the extreme rays of $\mathcal {M}_+$ are a set of vectors whose convex hull is $\mathcal {M}_+$ and such that they cannot be written as a linear combination of other vectors in $\mathcal {M}_+$, see (Rockafellar 1970)) are a basis for $\mathcal {M}$. Each extreme vector can be thought as the amount of the components of each of the substances of the system.

#### Lemma 2.4

Assume that the kinetic system $(\Omega , \mathcal {R}, \mathcal {K} )$ is conservative. Then the set of the extreme rays $\mathcal {B}$ of the positive cone $\mathcal {M}_+$ is a basis of $\mathcal {M}$.

For the proof of Lemma 2.4 we refer to Franco and Velázquez (2025a).

Let us define the subset of reactions $\mathcal {R}_s \subset \mathcal {R}$, obtained identifying each reaction *R* with the reversed reaction $- R$. More precisely, the set $\mathcal {R}_s \subset \mathcal {R}$ is defined as

$$ \mathcal {R}_s := \{ R \in \mathcal {R} : - R \notin \mathcal {R} \} \cup \{ R \in \mathcal {R} \setminus \{ R \in \mathcal {R} : - R \notin \mathcal {R} \} : \min {I(R) } < \min {F(R)} \}. $$

Hence we have that, if $R \in \mathcal {R}$ is such that $-R \notin \mathcal {R}$, then $R \in \mathcal {R}_s$. Instead if $R, - R \in \mathcal {R}$ only one of the two reactions belong to $\mathcal {R}_s$. We say that a kinetic system is *bidirectional* if for every $R \in \mathcal {R}$ we have that $- R \in \mathcal {R}$. If the kinetic system $(\Omega , \mathcal {R}, \mathcal {K} )$ is bidirectional, then $|\mathcal {R}_s| =r/2$.

#### Example 2.5

(*Non conservative system*) Consider the kinetic system given by

2.3 $$\begin{aligned} (1) \leftrightarrows (2)+(3), \quad (1)\leftrightarrows (3). \end{aligned}$$

The set of the conserved quantities is $\mathcal {M} = \operatorname {span} \{ (1,0,1)^T \}.$ Since $\mathcal {M} \cap \mathbb {R}_+^3 = \emptyset$, then the chemical network is not conservative.

#### Example 2.6

(*Conservative system*) Consider the kinetic system given by

$$ (1) + (1) \leftrightarrows (2)+(3), \quad (3)\leftrightarrows (4). $$

The set of the conserved quantities is

$$ \mathcal {M}= \left\{ m=(m_1, m_2 , m_3, m_4)^T \in \mathbb {R}^4 : m_3=m_4 , \ 2 m_1 = m_2+m_3 \right\} . $$

Notice that $(1,1,1,1)^T \in \mathcal {M} \cap \mathbb {R}_+^4$, hence the system is conservative. The basis of the extreme rays is

$$ \mathcal {B} = \{ (1,0,2,2)^T , (1,2,0,0)^T \}. $$

We can define the *cycles* of a chemical network $(\Omega , \mathcal {R})$.

#### Definition 2.7

(*Cycles of a kinetic system*) Let $(\Omega , \mathcal {R})$ be a chemical network. The space of the cycles of the chemical network $(\Omega , \mathcal {R})$ is defined as

$$ \mathcal {C} := \{ c \in \mathbb {R}^{|\mathcal {R}_s |}: \sum _{k=1}^{|\mathcal {R}_s | } c(k) R_k =0 \}. $$

A cycle *c* identifies a sequence of reactions (the reactions $$R_k $$ in $$\mathcal {R}_s $$ that are such that $$c(k) \ne 0$$ ) such that that when applied to a vector of concentrations have a null net effect.

#### Example 2.8

Consider the chemical network $(\Omega , \mathcal {R})$ associated with the chemical reactions

$$ (1) \leftrightarrows (2) \leftrightarrows (3) \leftrightarrows (1). $$

Here we have that $\mathcal {C} = \operatorname {span} \{ (1,1,1)^T \}$.

It is convenient to associate to a kinetic system $(\Omega , \mathcal {R}, \mathcal {K} )$ a system of ODEs as follows

2.4 $$\begin{aligned} \frac{d n (t) }{dt} = \sum _{ R \in \mathcal {R} } K_R \prod _{i\in I(R)} {(n_i)}^{-R(i)} R , \quad n(0)=n_0 \in \mathbb {R}_*^N. \end{aligned}$$

Here the solution $n:=(n_1, \dots , n_N)^T \in \mathbb {R}_*^N$ describes the change in time of the concentrations of substances in the network. For the purposes of this paper it is convenient to rewrite the system of ODEs corresponding to a bidirectional kinetic system as follows

2.5 $$\begin{aligned} \frac{dn(t)}{dt} = \sum _{ R \in \mathcal {R}_s } {J_R(n)} R , \quad n(0)= n_0 \in \mathbb {R}_*^N, \end{aligned}$$

where

2.6 $$\begin{aligned} J_R (n):= K_R \prod _{ j \in I(R) } {(n_j)}^{-R(j)} - K_{- R } \prod _{ j \in F(R) } {n_j}^{R(j)}. \end{aligned}$$

We refer to Lemma 2.10 in Franco and Velázquez (2025a) and to Feinberg (2019) for the details of this computation.

### Detailed balance property of kinetic systems

A bidirectional kinetic system satisfies the detailed balance property if at the steady state each reaction is balanced by its reverse reaction. We state the precise definition.

#### Definition 2.9

(*Detailed balance property*) A bidirectional kinetic system $(\Omega , \mathcal {R}, \mathcal {K} )$ satisfies the detailed balance property if there exists a $\overline{N} \in \mathbb {R}_+^N$ of (2.4) such that

2.7 $$\begin{aligned} K_R \prod _{i\in I(R)} {(\overline{N}_i)}^{-R(i) } = K_{-R} \prod _{i \in F(R) } {\overline{N}_i}^{R(i) } \ \text { for all } R\in \mathcal {R}_s. \end{aligned}$$

By the definition of detailed balance we have that $\overline{N}$ is such that $J_R(\overline{N}) =0$ for every $R \in \mathcal {R}$. In particular we have that $\overline{N}$ is a steady state of the system of ODEs (2.4). A consequence of the detailed balance property is that the steady states of (2.4) can be written as a function of the vector $E \in \mathbb {R}^N$, where *E* is the vector of the energies associated with each substance in the network.

#### Lemma 2.10

Assume that the kinetic system $(\Omega , \mathcal {R}, \mathcal {K})$ satisfies the detailed balance property. Then there exists a vector $E \in \mathbb {R}^N$ such that the equality

2.8 $$\begin{aligned} \mathcal {E}(R):= \log \left( \frac{\mathcal {K}(-R) }{\mathcal {K}(R) } \right) = \sum _{i \in \Omega } R(i) E(i) \end{aligned}$$

holds for every $R \in \mathcal {R}_s$. Moreover $N^* =(N^*_i)_{i=1}^N$ is a steady state of the system of ODEs (2.4) if and only if

2.9 $$\begin{aligned} N^*_i= e^{- E(i)}, \quad i \in \{ 1, \dots , L\} \end{aligned}$$

where $E \in \mathbb {R}^N$ is a solution of (2.8).

The proof of this lemma can be found in [Franco and Velázquez (2025a), Lemma 3.6]. A consequence of this lemma is that if the kinetic system $(\Omega , \mathcal {R}, \mathcal {K} )$ satisfies the detailed balance property, then equality (2.7) is attained at every steady state of the system of ODEs (2.4).

An important characterization of the kinetic systems that satisfy the property of detailed balance is the following.

#### Lemma 2.11

(Wegscheider criterion) Let $(\Omega , \mathcal {R}, \mathcal {K} )$ be a bidirectional kinetic system. It satisfies the detailed balance property if and only if, for every cycle $c \in \mathcal {C}$, it holds that

2.10 $$\begin{aligned} \prod _{ R_j \in \mathcal {R}_s } \left( \frac{K_{R_j}}{K_{-R_j}} \right) ^{c(j)} = 1. \end{aligned}$$

The proof of this lemma can be found in Wegscheider (1902) and in Feinberg (2019).

In the following, we refer to a solution to (2.8) as an *energy* of the kinetic system. The solution to (2.8) is not unique unless $$\mathcal {M} = \{0\} $$. Indeed if *E* is a solution to (2.8) and $$m \in \mathcal {M} $$, then also $$m+ E $$ is a solution to (2.8). Indeed for every $$R \in \mathcal {R} $$ it holds that

$$ \sum _{i \in \Omega } R(i) E(i) + \sum _{i \in \Omega } R(i)m (i) = \sum _{i \in \Omega } R(i) E(i) = \mathcal {E}(R). $$

We conclude with the definition of closed kinetic system, which is a system that does not exchange substances with the environment.

#### Definition 2.12

A kinetic system $(\Omega , \mathcal {R}, \mathcal {K} )$ is closed if it satisfies the detailed balance property, is conservative and is such that

2.11 $$\begin{aligned} I(R) \ne \emptyset \ \text { and } \ F(R) \ne \emptyset \text { for every } R \in \mathcal {R}. \end{aligned}$$

### The property of adaptation in signalling systems

**Fig. 2:**
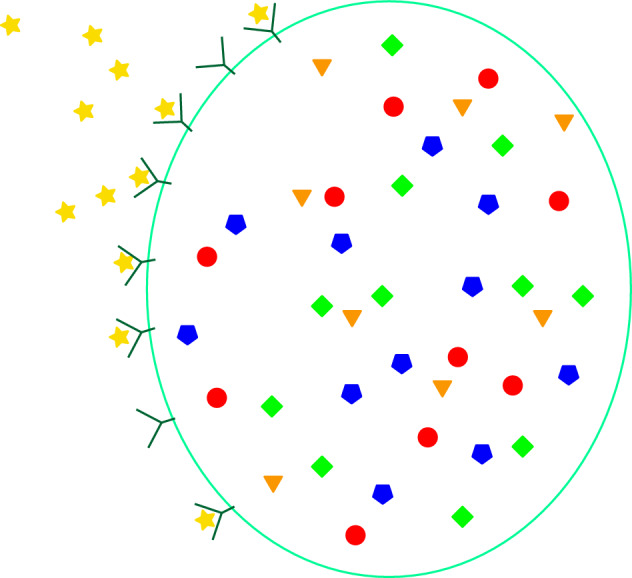
An example of signalling system that is conservative, i.e. it exchanges only the signal (yellow star) with the environment. The biological situation that we have in mind is the one of a cell that captures the signal using the receptors on the surface

In this section we give the definition of signalling systems. These are kinetic systems in which one of the concentration, the signal, changes in time according to a given function *f*. See Figure 2 for a visual representation of a signalling system. More precisely, a *signalling system* $(\Omega , \mathcal {R}, \mathcal {K}, n_1(t))$ is a kinetic system in which the concentration of the substance (1) is a given function of time. Therefore, the change in time of the concentrations of the substances of a signalling system is described by the following system of ODEs

2.12 $$\begin{aligned} \frac{d n (t) }{dt } = { \sum _{ R \in \mathcal {R}_s } J_R(n) R } + J^F, n_0 \in \mathbb {R}_*^N \end{aligned}$$

where $$ J^F(t):= e_1 J^F_1 (t)$$ and where $$J^F_1 (t)$$ is given and is such that $$n_1(t)=f(t) $$ and $$n_1(0)=f(0)$$. Moreover, we make the following assumptions on the function *f*(*t*). This assumption guarantees that the signal concentration converges to constant values sufficiently fast. Notice that the definition of signalling system that we use in this paper has analogies with the one used in Shinar and Feinberg (2010).

We now give a precise definition of the property of adaptation for signalling systems of the form (2.12). A signalling system satisfies the property of adaptation if there exists a substance $p \in \Omega$ whose concentration changes when the signal concentration changes, but returns to the initial concentration levels (i.e. $n_0^T e_p$) after this transient time.

#### Definition 2.13

(*The property of adaptation*) Consider the signalling system $(\Omega , \mathcal {R}, \mathcal {K}, n_1(t))$ where $n_1(t)=f(t)$ and *f* satisfies Assumption 2.14. Assume that *n* is the solution of equation (2.12) with initial datum $n_0=e^{- E} \in \mathbb {R}_*^{N}$ where *E* is an energy of $(\Omega , \mathcal {R}, \mathcal {K})$. We say that the signalling system satisfies the adaptation property with respect to the signal (1) and the product $p \in \Omega \setminus \{ 1 \}$ if it satisfies the following three conditions.

1. Let $\lim _ {t \rightarrow \infty } f(t) = \overline{n}_1$. Then, there exists a unique steady state $N[ \overline{n}_1] \in \mathbb {R}_+^N$, that might depend on $\overline{n}_1$, such that
  $$ \lim _{t \rightarrow \infty } n(t) = N[ \overline{n}_1]. $$
2. The steady state $N[\overline{n}_1]$ satisfies $N[ \overline{n}_1](p)=n_0^T e_p$, in particular it is independent on $\overline{n}_1$.
3. It holds that $\sup _{ t>0} | n_p (t ) - n_0^T e_p | > 0.$

The first property guarantees that the system of ODEs (2.12) converges to a steady state as time tends to infinity, this steady state could depend on $\overline{n}_1$. The second property guarantees that the concentration of the substance (*p*) at this steady state does not depend on the limiting value of the signal $\overline{n}_1$. In particular the concentration of the substance (*p*) is given by the initial concentration of substance (*p*), i.e. $n_0^T e_p$. This guarantees that if we assume that the system is initially at steady state and we perturb it by changing the concentration of the signal, then, as time tends to infinity, the concentration of (*p*) converges to the steady state value that it had before the change in the signal. The fact that the concentration of product (*p*) at the steady state does not depend on $\overline{n}_1$ does not contradict the fact that the set of all the concentrations at steady state could depend on $\overline{n}_1$. The property 3. in the definition of adaptation guarantees that the system reacts to changes of the signal. We refer to Figure 1 for a graphical representation of the property of adaptation.

Notice that we are not assuming that the kinetic system $\frac{d n (t) }{dt } = \sum _{ R \in \mathcal {R}_s } J_R(n) R$ associated to the signalling system (2.12) satisfies the property of detailed balance or that it is conservative. However we have proven in Franco and Velázquez (2025a) that the set of reactions and substances associated to (2.12) can be extended to a kinetic system (with more substances and reactions) having the property that if we freeze some of the concentrations at constant values the system reduces to (2.12). Following (Franco and Velázquez 2025a) we refer to this larger system as a completion of (2.12). This completion allows to rewrite (2.12) as a thermodynamically admissible signalling system (cf. (1.3)). Therefore if (2.12) has the robust adaptation property then also the equivalent completed thermodynamically admissible system has the robust property of adaptation. We remark that the adaptation property of (2.12) remains valid if we change the values of the frozen concentrations in the completed system. This is due to the fact that the new chemical rates of the system of the form (2.12) obtained with the new values of the frozen quantities depend continuously on the values of the frozen concentrations (and we assumed that (2.12) has robust adaptation). Then the procedure described in the previous paragraph provides a way of obtaining thermodynamically admissible systems having robust adaptation taking as starting point any model of the form (2.12) describing bidirectional reactions.

A key step in the proof of Proposition 3.5 is to introduce a large class of signalling systems that can be modeled as in (2.12) for which detailed balance holds. This will be made in Section 3.2. To conclude this section we prove an auxiliary result that will be required later, namely that for systems of the form (2.12) satisfying the detailed balance condition and a signal converging sufficiently fast to constant values the solution converges to a steady state.

#### Assumption 2.14

(*Assumption on the signal*) We assume that $$f: \mathbb {R}_* \rightarrow \mathbb {R}_* $$ is a continuously differentiable function such that there exists a $$r >0 $$ such that $$|\frac{d}{dt}\log (f)|< e^{ - rt } $$ and

2.13 $$\begin{aligned} \lim _{t \rightarrow \infty } f(t) = \overline{n}_1>0. \end{aligned}$$

#### Proposition 2.15

Assume that $(\Omega , \mathcal {R}, \mathcal {K})$ satisfies the detailed balance property and is such that $\mathcal {M} \ne \{ 0\}$. Let $n_0 \in \mathbb {R}_*^N$. Assume that the function *f*(*t*) satisfies Assumption 2.14 and is such that $f(0)=n_0(1)$. Assume moreover that the system of ODEs (2.12) admits only positive steady states. Then the solution of the system of ODEs (2.12) is such that

2.14 $$\begin{aligned} \overline{J}^F_1:=\int _0^\infty J_1^F (t) dt < \infty . \end{aligned}$$

Moreover, for every $n_0\in \mathbb {R}^N$ we have that $\lim _{t \rightarrow \infty } n(t)= e^{-E}$ where *E* is the unique energy of $(\Omega , \mathcal {R}, \mathcal {K})$ that is such that

2.15 $$\begin{aligned} m^T e^{- E }- m^T n_0 = m^T \overline{J}^F_1 \text { for every } m \in \mathcal {M}. \end{aligned}$$

#### Proof

Notice that there exists an energy $$E_0 $$ of the kinetic system $$(\Omega , \mathcal {R}, \mathcal {K} ) $$ that is such that $$E_0(1)= - \log (n_0(1))= - \log (f(0))$$. Indeed since the kinetic system satisfies the detailed balance property we know that there exists an $$\overline{E} $$ such that (2.8) holds for every $$R \in \mathcal {R}_s$$. Assume that $$ \overline{E}(1) \ne - \log (n_0(1))= - \log (f(0))$$. Without loss of generality we assume that there exists a $$m \in \mathcal {M} $$ that is such that $$m(1)>0$$. Then we can define $$E_0$$ as

$$ E_0=\overline{E} - m \frac{ \overline{E}+ \log (n_0) }{m(1)} $$

where $m \in \mathcal {M}$ is such that $m(1) >0$.

Let us fix a conservation law $m \in \mathcal {M}$ such that $m(1)>0$. Then we can define the function $E^s: \mathbb {R}_* \rightarrow \mathbb {R}^N$ as

$$ E^s(t):=E_0 + \frac{m }{m(1) } \left[ \log (n_0(1)) - \log (f(t)) \right] . $$

We can then define the function $n^s$ as $n^s(t):= e^{- E^s(t)}.$ By the definition of $E^s$ we have that

$$ \frac{d }{dt} n_j^s(t)= - n_j^s(t) \frac{d }{dt} E_j^s(t)= \frac{m(j) }{m(1)} n_j^s(t) \frac{1}{f(t)} \frac{d f(t)}{dt}, \quad \forall j \in \{1, \dots , N\}. $$

Moreover, notice that since *f* is assumed to be bounded, then we also have that $n^s$ is bounded. Let us consider the function $F_r: \mathbb {R}_*^N \rightarrow \mathbb {R}$ defined as

$$ F_r(n) := \sum _{\Omega \setminus \{ 1\} } n_j \left( \log \left( \frac{n_j}{n_j^s} \right) -1 \right) . $$

We have that

$$\begin{aligned} \partial _t F_r(n) = \sum _{\Omega \setminus \{ 1\} } \partial _t n_j \log \left( \frac{n_j}{n_j^s} \right) - \sum _{\Omega \setminus \{ 1\} } \frac{n_j}{n_j^s}\frac{d}{dt} n^s_j = \mathcal {D}_r (n) - \sum _{\Omega \setminus \{ 1\} } \frac{m(j)}{m(1)}n_j (t) \frac{1}{f(t)} f'(t) \end{aligned}$$

where we have that

$$ \mathcal {D}_r (n) := \sum _{ R \in \mathcal {R} } K_R \prod _{ i \in \Omega \setminus \{1\} } n_i^{- R(i) } \log \left( \prod _{j \in \Omega \setminus \{ 1 \} } \left( \frac{n_j}{n^s_j} \right) ^{R(j) } \right) \left( 1- \prod _{k \in \Omega \setminus \{1 \} } \left( \frac{n_k}{n^s_k} \right) ^{R(k) } \right) \le 0. $$

As a consequence, using the fact that *f*, $$f'$$ and $$n_s $$ are bounded we notice that for every $$t>0$$

$$ \partial _t F_r(n) \le - \sum _{\Omega \setminus \{ 1\} }\frac{m(j)}{m(1)}n_j(t)\frac{1}{f(t)} f'(t) < c_1 e^{-rt} \sum _{\Omega \setminus \{ 1\} }n_j(t) \le c_1 e^{-rt} \left( c_2 + F_r(n) \right) $$

for some constants $$c_1, c_2 >0 $$. By Grönwall’s inequality we deduce that for every $$t >0 $$ we have that $$F_r(t) < \infty $$. Moreover, we deduce also that $$\sup _{ t >0 } F_r(n) < \infty $$, hence $$ \sup _{ t >0 } n (t) < \infty $$. On the other hand notice that since there exists a $$m \in \mathcal {M} $$ such that $$m(j) >0 $$ for every $$j \in \Omega $$ we deduce that

$$ m^T n (t) - m^T n_0 = m(1) \int _{0}^t J_1^F (s) ds $$

taking the supremum as $t \rightarrow \infty$ we deduce that $\int _{0}^\infty J_1^F (s) ds < \infty$. Moreover we deduce that $\lim _{ t \rightarrow \infty } n (t)=n(\infty )$ where $n(\infty ) \in \mathbb {R}_*^N$. From this we deduce that since $\int _0^\infty \mathcal {D}_r(n(s) ) ds < \infty$ and $\lim _{t \rightarrow \infty } \mathcal {D}_r(n) < \infty$, then $\lim _{ t \rightarrow \infty } \mathcal {D}_r(n)=0$. By continuity this implies that $\lim _{ t \rightarrow \infty } n(t) = \lim _{ t \rightarrow \infty } n^s(t)= e^{- E}$ where *E* is the unique energy of $(\Omega , \mathcal {R}, \mathcal {K} )$ such that $E_1 =- \log ( \overline{n}_1 )$ that satisfies (2.15). $\square$

#### Example 2.16

(*Signalling system*) Consider the following set of reactions

$$ (1) \leftrightarrows (2),\quad (1) \leftrightarrows (3), \quad (2)\leftrightarrows (3) + (4). $$

We assume that all the reactions take place at rate 1 except for the reactions $$ (2) \rightarrow (1) $$ and $$(3) \rightarrow (1) $$ that take place at the same rate $$\alpha >0$$. We can define a kinetic system $$(\Omega , \mathcal {R}, \mathcal {K} ) $$ corresponding to this set of chemical reactions. Moreover, we stress that the substance (1) is connected with the substance (4). Assume that the concentration of (1) is given i.e. $$n_1(t)=f(t)$$ for a function *f* that satisfies Assumption 2.14. Then the evolution of $$(n_2,n_3, n_4) $$ is given by

$$\begin{aligned} \frac{d n_2 }{dt }&=f(t)- (\alpha +1) n_2 + n_3 n_4 \\ \frac{d n_3 }{dt }&=f(t) - \alpha n_3 + n_2 - n_3 n_4 \\ \frac{d n_4 }{dt }&= n_2 - n_3 n_4. \end{aligned}$$

The above system of ODEs defines a signalling system of the form (2.12).

## Analysis of the adaptation property for some classes of signalling systems

In this section we study the property of adaptation for signalling systems. As explained in the introduction, the property of adaptation is an important property of several biological systems.

### Response of connected systems to changes in the signal concentration

For the purposes of this section it is convenient to consider the following graph associated with a kinetic system $(\Omega , \mathcal {R} )$. The graph $\mathcal {G}_\Omega =(V, E)$ has vertices

$$ V:= \Omega $$

and edges

$$ E:=\{(\alpha , \beta ) \in V : \exists R \in \mathcal {R} \text { with } R(\alpha ) R(\beta ) \ne 0 \}. $$

#### Definition 3.1

Let $(\Omega , \mathcal {R}, \mathcal {K}, n_1(t) )$ be a signalling system. We say that two substances $i, j \in \Omega$, are connected if there exists a walk in $\mathcal {G}_\Omega$ that connects them. The signalling system is connected if every couple of elements $i, j \in \Omega$ is connected.

The following example shows that the fact that the substance $1\in \Omega$ is connected with $p \in \Omega \setminus \{ 1\}$ is not a sufficient condition for the property 3 in Definition 2.13 to hold.

#### Example 3.2

(*Connected system with detailed balance and with no response*) Consider the signalling system introduced in Example 2.16 with initial condition $$n_0 \in \mathbb {R}_*^3$$ given by $$n_0=(\frac{f(0) }{\alpha },\frac{f(0) }{\alpha },1)^T$$. The underlying kinetic systems connected in the sense of Definition 3.1. Notice that a steady state of the above system must be of the form $$ (N_1, N_1, 1)$$. Moreover,

$$\begin{aligned} \frac{d \left( n_2 - n_4 n_3 \right) }{dt }=\frac{d n_2 }{dt } - n_3 \frac{d n_4 }{dt } - n_4 \frac{d n_3 }{dt } = F(n_2, n_3, n_4) \end{aligned}$$

where

$$ F(n_2, n_3, n_4) : = (1- n_4) f(t) - (1+\alpha ) (n_2 - n_4 n_3) - (n_4+n_3 ) (n_2 - n_4n_3 ). $$

Notice that for every $$N_1 >0 $$ we have that $$F(N_1, N_1,1)=0$$.

We now assume that $$f(t) \rightarrow \overline{n}_1 \ne f(0)$$ as $$t \rightarrow \infty $$. Hence we assume that the signal changes in time. However $$n_2 (t) - n_4(t) n_3 (t) =0$$ for every $$t>0 $$ because $$F(n_2, n_3, n_4) =0$$ for every $$t >0$$. Hence $$n_4(t)=1 $$ for every $$t >0 $$. As a consequence there is no change in the concentration of the substance (4) after changes of the signal concentration. Therefore, the property 3 in the definition of adaptation does not hold even if the network is connected.

In Example 3.2 the product and the signal are connected, but changes in the signal do not produce changes in the product concentration. However, the parameters in Example 3.2 are fine tuned. Indeed, if we perturb the rates of the reactions taking place in the network, then we obtain that the property 3 in Definition 2.13 holds. This motivates us to prove the following theorem that states that if the kinetic system $$(\Omega , \mathcal {R}, \mathcal {K}) $$ satisfies the detailed balance condition and is connected, then the property 3 in Definition 2.13 can fail only in an unstable manner, i.e. it fails only for fine tuned reaction rates. Before stating the theorem we present an additional assumption that the function *f*, describing the change in time of the signal, should satisfy in order for the theorem to be valid. In particular the signal concentration $$n_s=f $$ must change in the following way for small times

3.1 $$\begin{aligned} f (t) - n_0(1) \sim t \text { as } t \rightarrow 0^+. \end{aligned}$$

A function *f* that satisfies both (3.1) and Assumption 2.14 is called *admissible signal*.

#### Theorem 3.3

(Non-trivial response) Assume that $(\Omega , \mathcal {R}, \mathcal {K})$ satisfies the detailed balance property. Assume that $1, p \in \Omega$ are connected. Then for every $\delta >0$ there exists a rate function $\overline{\mathcal {K}}: \mathcal {R} \rightarrow \mathbb {R}_+$ such that

1. $$\Vert \mathcal {K} - \overline{\mathcal {K}} \Vert < \delta $$;
2. $(\Omega , \mathcal {R}, \overline{\mathcal {K}})$ satisfies the detailed balance property;
3. the solution $\overline{n}$ to the system of ODEs (2.12) induced by $(\Omega , \mathcal {R}, \overline{\mathcal {K}} )$ for an admissible signal satisfies property 3 in Definition 2.13.

Before the proof of Theorem 3.3 we present an example of a closed kinetic system that is such that the graph $\mathcal {G}_\Omega$ is connected and the property 3 holds generically, i.e. if property 3 does not hold then it is possible to construct a perturbation of the reaction rates that is such that the perturbed kinetic system satisfies the property 3, i.e. the system reacts to changes in the signal. The purpose of this example is also to illustrate the proof of Theorem 3.3 in a simpler situation.

#### Example 3.4

Consider the chemical network corresponding to the following reactions

$$ (1) \leftrightarrows (2)+(3), \quad (2) \leftrightarrows (3)+(4). $$

Let $R_1=(1,-1,-1,0)^T$ and $R_2=(0,1,-1,-1)^T$. This chemical network is connected. Moreover, we assume that

$$ K_{- R_1} = K_{R_1} e^{E(1)- E(2) - E(3) }, \text { and } K_{- R_2} = K_{R_2} e^{E(2) - E(3) - E(4) } $$

for some vector $$E \in \mathbb {R}^4$$. In this way we assume that the detailed balance property holds by construction. In particular the energies of the reaction $$R_1 $$ and $$R_2$$ are given by

$$ \mathcal {E} (R_1) = E(1)- E(2) - E(3), \text { and } \ \mathcal {E} (R_2)= E(2) - E(3) - E(4). $$

To simplify the notation we indicate with $$K_1 $$ the rate $$K_{R_1} $$ and with $$K_2$$ the rate $$K_{R_2} $$. We can write the system of ODEs associated with the kinetic system

$$\begin{aligned} \frac{d n_1}{d t}&=K_1 \left( n_2 n_3 - e^{\mathcal {E}(R_1) } n_1 \right) \\ \frac{d n_2}{d t}&=K_1 \left( e^{\mathcal {E}(R_1) } n_1 - n_2 n_3\right) + K_2 \left( n_3 n_4 - e^{\mathcal {E}(R_2) } n_2 \right) \\ \frac{d n_3}{d t}&=K_1 \left( e^{\mathcal {E}(R_1) } n_1 - n_3 n_3\right) + K_2 \left( e^{\mathcal {E}(R_2) } n_2 - n_3 n_4 \right) \\ \frac{d n_4}{d t}&= K_2 \left( e^{\mathcal {E}(R_2) } n_2 - n_3 n_4 \right) \end{aligned}$$

We linearize the system around the steady state $N=e^{- E}$. In particular we consider $n_k =(1+ \varphi _k ) e^{- E(k) }$ with $\varphi _1 (t) = t$. Hence

$$\begin{aligned} \varphi _1 (t)&= t \\ \frac{d \varphi _2}{d t}&=K_1 e^{- E(3) } \varphi _1 + l.o.t. \\ \frac{d \varphi _3}{d t}&=K_1 e^{- E(2) } \varphi _1 + l.o.t. \\ \frac{d \varphi _4}{d t}&= K_2 e^{-E(3) }\left( \varphi _2 - \varphi _3 \right) + l.o.t. \end{aligned}$$

As a consequence we obtain that $\varphi _2 \sim \frac{K_1}{2} e^{- E(3) } t^2$ as $t \rightarrow 0$ and $\varphi _3 =\frac{K_1}{2} e^{- E(2) } t^2$ as $t \rightarrow 0$. Therefore

$$ \varphi _4 (t)\sim \frac{K_1 K_2 e^{- E(3)}}{6} \left( e^{- E(3)} - e^{-E(2)} \right) t^3 \quad \text { as } t \rightarrow 0. $$

As a consequence we have that $\varphi _4(t) \ne 0$ for sufficiently small times, except if $E(3)=E(2)$, i.e. unless the reaction rates are fine tuned, we have that the concentration of substance (4) changes in time.

#### Proof of Theorem 3.3

The idea of the proof is the following. Initially the system is at steady state. We consider a small perturbation of the substance (1) and study the evolution of the concentration of (*p*) for small times. Since we are considering a small perturbation and we are considering small times we can study the linearization of the system of ODEs (2.12). As a second step we define a hierarchy on the elements $\Omega \setminus \{ 1\}$ that are connected to the substance (1). In particular the hierarchy is based on the distance of the elements from (1). The distance here has to be understood as distance in the graph $\mathcal {G}_\Omega$. The reason why we introduce this hierarchy is that the concentration of substances that are closer to (1) change faster than the concentration of substances that are far from the signal (1). We will make this idea rigorous in Step 2. Once the hierarchy is defined we can prove that, unless the parameters are fine tuned, we have a response in each point in $\Omega \setminus \{1\}$ that is connected with the substance (1). This is done in Step 3.

**Step 1: Linearization.**

Since the kinetic system satisfies the detailed balance property we have that $$n_0=e^{-E} $$ where *E* is an energy of $$(\Omega , \mathcal {R}, \mathcal {K})$$, i.e. *E* is a solution to (2.8). We can write the solution *n* to the signalling system (2.12) as $$n= e^{- E} (1 + \overline{\varphi }) $$ where, since by assumption $$n_1 - n_1(0) \sim e^{E(1)} t $$ as $$t \rightarrow 0$$, we have that $$\overline{\varphi }_1(t) \sim e^{E(1)} t $$ as $$ t \rightarrow 0 $$. Moreover by the continuity in time of *n* we have that $$\Vert \overline{\varphi }(t) \Vert \rightarrow 0 $$ as $$ t \rightarrow 0$$. Since the detailed balance property holds we deduce that for every reaction $$R \in \mathcal {R} $$ it holds that

$$ \log \left( \frac{K_{-R}}{K_R} \right) =\mathcal {E}(R)= \sum _{i \in \Omega } R(i) E(i). $$

Substituting this in (2.5) we deduce that

3.2 $$\begin{aligned} J_R(n) =K_R \prod _{i \in I(R)} (n_i)^{- R(i) } \left( 1- \prod _{i\in \Omega } n_i^{ R(i) } e^{ R(i) E(i) } \right) , \forall n \in \mathbb {R}_*^N. \end{aligned}$$

Using the fact that $n = e^{- E} ( 1 + \overline{\varphi })$ we obtain that

$$\begin{aligned} J_R(n)&=K_R \prod _{i \in I(R)} e^{ E(i) R(i)} (1+\overline{\varphi }_i)^{- R(i)} \left( 1- \prod _{k \in \Omega } (1+\overline{\varphi }_k{R(k)})\right) + o(\Vert \overline{\varphi }\Vert ) \\&=- K_R \prod _{i \in I(R)} e^{ E(i) R(i)} (1-\overline{\varphi }_i R(i) ) \prod _{k \in \Omega } \overline{\varphi }_k{R(k)} + o(\Vert \overline{\varphi }\Vert ) \\&= - K_R e^{ \sum _{i \in I(R)} E(i) R(i)} \sum _{k \in \Omega } \overline{\varphi }_k{R(k)} + o(\Vert \overline{\varphi }\Vert ). \end{aligned}$$

Therefore equation (2.6) implies that

$$\begin{aligned} e^{-E(\ell ) } \frac{d \overline{\varphi }_\ell }{ dt } = \sum _{R \in \mathcal {R}_s } R(\ell ) J_R(n ) =- \sum _{R \in \mathcal {R}_s } K_R \prod _{i \in I(R)}e^{ E(i) R(i)} R (\ell )\sum _{k \in \Omega }\overline{\varphi }_k{R(k)} + o(\Vert \overline{\varphi }\Vert ). \end{aligned}$$

Notice that to prove that $\sup _{ t>0} |\overline{\varphi }_p (t) |>0$ it is enough to show that $\sup _{t \in (0,T]} |\varphi _p(t) |>0$ for every $T>0$, where $\varphi$ is the solution to the following linearized problem

3.3 $$\begin{aligned} {\left\{ \begin{array}{ll}\varphi _1 (t) & = e^{ E(1)} t \\ \frac{d \varphi _\ell }{ dt } & = \sum _{j \in \Omega } A_{\ell j } \varphi _j \quad \ell \in \Omega \setminus \{ 1 \} \end{array}\right. } \end{aligned}$$

where

$$ A_{\ell j } = - e^{E(\ell ) } \sum _{ R \in \mathcal {R}_s} R(j) R(\ell ) K_R \alpha _R \ \text { for } \ell \in \Omega \setminus \{ 1 \} \text { and } j \in \Omega $$

and where we are using the notation

3.4 $$\begin{aligned} \alpha _R= \prod _{ i \in I(R) } e^{E(i) R(i)}. \end{aligned}$$

**Step 2: Hierarchy of responses.**

In order to prove that for every $$T>0 $$ we have that $$\sup _{t \in (0, T] } |\varphi _p(t) | >0$$ we construct a hierarchy of elements of $$\Omega $$ based on their distance from the signal 1. To this end we define the sets $$S_0:=\{1\}$$ and

$$ S_n:= \{ i \in \Omega \setminus S_{n-1}: \exists R \in \mathcal {R} \text { and } k \in S_{n-1} \text { s.t. } R(i) R(k) \ne 0 \} $$

for every $n \in \{1, \dots , L-1 \}$ where *L* is the minimal length of the walks connecting (1) with (*p*) in the graph $\mathcal {G}_\Omega$. Moreover we assume that $S_L:=\{ p \}$.

We are going to prove that for every $\ell \in S_{n}$, with $n \in \{ 1, \dots , L \}$, we have that

3.5 $$\begin{aligned} \varphi _\ell (t)= c_{ n +1, \ell }(\mathcal {K}, E) t^{n+1} + o(t^{n+1}) \end{aligned}$$

where the constant $c_{ n } (\mathcal {K}, E) \ge 0$ is given by

3.6 $$\begin{aligned}&c_{ n, \ell }(\mathcal {K}, E ):= e^{E(1)} \frac{1}{n!} \sum _{j_0 \in S_0} \sum _{ j_{1} \in S_{1} } A_{j_1 j_0} \sum _{ j_{2} \in S_{2} } A_{j_2 j_1 } \dots \sum _{ j_{n-2} \in S_{n-2} } A_{j_{n-2} j_{n-3} }\nonumber \\&\sum _{ j_{n-1} \in S_{n-1} } A_{j_{n-1} j_{n-2} } A_{ \ell j_{n-1}} =e^{E(1)} \frac{1}{n!} \sum _{j \in X_n} a(j) \end{aligned}$$

where $$X_n = S_0 \times S_1 \times \dots \times S_{n-1} \times \{ \ell \} $$, therefore $$j =(j_0, j_1, \dots , j_{n-1}, j_n) $$ with $$j_0=1 $$, $$j_n=\ell $$ and $$j_i \in S_i $$ for every $$i \in \{ 1, \dots , n-1\} $$. Moreover for $$j \in X_n $$ we have

$$ a(j):= \prod _{i=1}^n A_{j_i, j_{i-1} }. $$

In other words $$c_{n, \ell } (\mathcal {K}, E)$$ is defined inductively as follows. We assume $$c_{1, \ell } (\mathcal {K}, E) = e^{E(1)} A_{\ell 1 }$$ for every $$\ell \in S_1 $$ and that $$c_{n, \ell } (\mathcal {K}, E) = \sum _{j_{ n-1} \in S_{n-1}} c_{n-1, j_{n-1}}(\mathcal {K}, E) A_{\ell j_{n-1}}$$ for every $$n > 1 $$ and $$\ell \in S_n$$.

We prove now (3.5). We start by proving the equality for $n =1$. More precisely we prove that

3.7 $$\begin{aligned} \varphi _\ell (t)= c_{1, \ell } (\mathcal {K} , E) t^2+ o(t^2) \quad \forall \ell \in S_1, \text { as } t \rightarrow 0^+. \end{aligned}$$

The system of equations (3.3) implies that

$$ \frac{d \varphi _\ell }{dt } = \sum _{j \in \Omega } A_{\ell j } \varphi _j = A_{\ell 1 } \varphi _1+ \sum _{j \in \Omega \setminus \{ 1 \} } A_{\ell j } \varphi _j =t e^{E(1)} A_{\ell 1 } + \sum _{j \in \Omega \setminus \{ 1 \} } A_{\ell j } \varphi _j. $$

By the definition of $c_{1, \ell } (\mathcal {K}, E)$ we have

$$ c_{1, \ell } (\mathcal {K} , E) = \frac{1}{2} e^{E(1) } A_{\ell 1 }, \quad \forall \ell \in S_1. $$

As a consequence

$$ \varphi _\ell (t)= c_{1, \ell }(\mathcal {K} , E) t^2 + \sum _{j \in \Omega \setminus \{ 1 \} } A_{\ell j } \int _0^t \varphi _j(s) ds, \quad \forall \ell \in S_1. $$

Since for every $$j \in \Omega \setminus (\{1\} \cup S_1)$$ it holds that $$\varphi _j (t)=o(t) $$ as $$t \rightarrow 0$$ we have that (3.7) holds. If $$L=1 $$, then equality (3.5) follows for $$\ell \in S_1$$.

Assume that $L > 1$. We now prove (3.5) by induction. Therefore let us assume that for every $k \in S_{n-1}$ it holds that

$$ \varphi _k (t)= c_{n-1, k}(\mathcal {K} , E) t^{n} + o(t^{n}). $$

The equation for $\ell \in S_n$ is the following

$$\begin{aligned} \frac{d \varphi _\ell }{ dt } = \sum _{ j \in S_{n -1} } A_{\ell j } \varphi _j + \sum _{i=n}^{L} \sum _{ j \in S_i } A_{\ell j } \varphi _j = \sum _{ j \in S_{n -1} } A_{\ell j } c_{n-1, j}(\mathcal {K} , E) t^{n} + o(t^{n}) \quad t \rightarrow 0^+. \end{aligned}$$

In the equality above we have used the fact that $\varphi _j (t) = o(t^n)$ as $t \rightarrow 0$ if $j \in S_n$. Using the definition of $c_{n, \ell }$ and integrating in time the equality above we obtain equality (3.5) for every $n \in \{ 0, \dots , L \}$.

**Step 3: There exists a rate function** $\overline{ \mathcal {K}}$ **as in the statement of the theorem and such that** $c_{L,p} (\overline{\mathcal {K}}, \overline{E}) \ne 0$.

In order to prove step 3 it is useful to define the following set of reactions

3.8 $$\begin{aligned} \Gamma _{n, n+1} := \left\{ R\in \mathcal {R}_s : \exists k \in S_{n+1}, \ell \in S_{n} \text { s.t. } R(k) R(\ell ) \ne 0 \right\} \text { for every } n \in \{ 0, \dots , L \}. \end{aligned}$$

We stress that if $$n_1 \ne n_2 $$, then $$ \Gamma _{n_1, n_1+1} \cap \Gamma _{n_2, n_2+1} = \emptyset .$$ Indeed, assume by contradiction that there exists $$R \in \mathcal {R}_s $$ such that $$R \in \Gamma _{n_1,n_1+1} \cap \Gamma _{n_2,n_2+1} $$ where $$n_1\ne n_2 $$, where without loss of generality we can assume that $$n_1 < n_2$$. This implies that there exist $$k_1 \in S_{n_1} $$ and $$\ell _1 \in S_{n_1+1} $$ such that $$R(k_1) R(\ell _1) \ne 0$$ and there exist $$k_2 \in S_{n_2} $$ and $$\ell _2 \in S_{n_2+1} $$ such that $$R(k_2) R(\ell _2) \ne 0$$. Notice that by the definition of the sets $$S_n $$ we have that since $$\ell _2 \in S_{n_2+1} $$, then $$\ell _2 \notin S_{n_1+1} $$. On the other hand, notice that $$R(\ell _2) R(k_1) \ne 0$$, which implies that $$\ell _2 \notin S_{n_1+1} $$. This is a contradiction, hence the sets $$\{ \Gamma _{n, n+1} \}_{n=0}^L$$ are disjoint sets.

The notation above allows us to rewrite the formula for some of the elements of the matrix *A*, i.e. for the elements $A_{\ell j }$ with $j \in S_n$ and $\ell \in S_{n+1}$. Indeed we have that

$$ A_{\ell j } = - e^{E(\ell ) } \sum _{ R \in \Gamma _{n, n+1} } R(j) R(\ell ) K_R \alpha _R \ \text { for } \ell \in S_{n+1} \text { and } j \in S_n \text { and } 0 \le n \le L-1, $$

where we recall that $\alpha _R$ is given by (3.4). We substitute this formula for $A_{\ell j }$ in (3.6). In this way we obtain that

$$\begin{aligned} c_{L, p } (\mathcal {K} , E)&= e^{E(1)} \frac{1}{L!} \sum _{j \in X_L}\prod _{i=1}^L A_{j_i j_{i-1}}= e^{E(1)} \frac{(-1)^L}{L!} \sum _{j \in X_L} \prod _{i=1}^L e^{E(j_i) } \sum _{R_i \in \Gamma _{i-1,i}} \alpha _{R_i} K_{R_i} R_i(j_i) R_i(j_{i-1}) = \\&=(-1)^L \frac{1}{L!} \sum _{ j \in X_L } \sum _{ R_1 \in \Gamma _{0, 1} } \sum _{ R_2 \in \Gamma _{ 1, 2 } } \dots \sum _{ R_{L-2} \in \Gamma _{L-3,L-2} } \sum _{ R_{L-1} \in \Gamma _{ L-2, L-1 } } \sum _{ R_{L} \in \Gamma _{ L-1, L } } e^{\sum _{i=0}^{L} E(j_i) } \\&\prod _{i=1}^L \alpha _{R_i} K_{R_i} R_i(j_{i-1}) R_1(j_i) =\sum _{j\in X_L} \sum _{r \in Y_L} v(r,j) \end{aligned}$$

where $Y_L:=\Gamma _{0,1} \times \Gamma _{1,2} \times \dots \times \Gamma _{L-2,L-1} \times \Gamma _{L-1,L}$ and where $r =(R_1, R_2, \dots , R_{L-1}, R_L)$ with $R_i \in \Gamma _{i-1,i}$ for $i\in \{ 1, \dots , L\}$. Moreover given $j \in X_L$ and $r \in Y_L$ we have that

$$\begin{aligned} v(r, j)&:= e^{\sum _{i=0}^{L} E(j_i) } \prod _{i=1}^L \alpha _{R_i} K_{R_i} R_i(j_{i-1}) R_i(j_i) =e^{\sum _{i=0}^{L} E(j_i) } \prod _{i=1}^L \alpha _{R_i} K_{R_i} R_i(j_{i-1}) R_i(j_i) \\&= e^{\sum _{i=0}^{L} E(j_i) } \prod _{i=1}^L e^{\sum _{k \in I(R_i)} E(k) R_i(k)} K_{R_i} R_i(j_{i-1}) R_i(j_i) . \end{aligned}$$

Notice that we have two possibilities, either $$c_{L, p } (\mathcal {K}, E) \ne 0$$ or $$c_{L, p } (\mathcal {K}, E)= 0$$. If $$c_{L, p } (\mathcal {K}, E) \ne 0$$ then we have that (3.5) implies that for every $$T>0 $$ it holds that $$\sup _{ t \in [0, T] } |\varphi _p(t)|>0$$. Hence the statement of the theorem follows.

Assume instead that $c_{L, p } (\mathcal {K}, E) =0$. We want to construct a perturbation $\overline{\mathcal {K}}$ of the rate function $\mathcal {K}$ that is such that $c_{L, p }(\overline{\mathcal {K}}, \overline{E}) \ne 0$. Moreover, we want that the perturbed kinetic system $(\Omega , \mathcal {R}, \overline{\mathcal {K}} )$ satisfies the detailed balance property. To construct this perturbation we argue as follows. We select the shortest path $\pi$ that connects (1) with (*p*) in the graph $\mathcal {G}_\Omega$. This path $\pi$ identifies a sequence of reactions $\{ \overline{R}_i\}_{i=1}^L$ and a sequence of points $\{ j_i^\pi \}_{i=0}^{L}$ in $\Omega$. Notice that by definition we have that $\overline{R}_i \in \Gamma _{i-1,i}$ for every $i \in \{ 1, \dots , L \}$. We modify the rates of the reactions $\{ \overline{R}_i\}_{i=1}^L$. More precisely we consider the rate function $\overline{\mathcal {K}}: \mathcal {R} \rightarrow \mathbb {R}_+$ defined as

3.9 $$\begin{aligned} \overline{\mathcal {K}} (R) := {\left\{ \begin{array}{ll} \mathcal {K}(R) & {\text { if }} R \notin \{ \overline{R}_i\}_{i=1}^L \text { and } -R \notin \{ \overline{R}_i\}_{i=1}^L \\ \mathcal {K}(R) + \delta _R & {\text { if }} R \in \{ \overline{R}_i\}_{i=1}^L. \end{array}\right. } \end{aligned}$$

Finally, we assume that

$$ \overline{\mathcal {K}} ( -R) := \frac{\overline{\mathcal {K}} (R) \mathcal {K} (-R)}{ \mathcal {K} ( R) } \exp \left( \sum _{i \in \pi } R(i) \varepsilon _i\right) \text { if } R \in \{ \overline{R}_i\}_{i=1}^L $$

for some $\varepsilon _i >0$, where $i \in \pi$. The kinetic system $(\Omega , \mathcal {R}, \overline{\mathcal {K}} )$ satisfies the detailed balance property by construction. Indeed, by construction, we have that

$$ \overline{\mathcal {E} }(R) := \log (\overline{\mathcal {K}} (- R)/\overline{\mathcal {K}} (R)) = \log ( {\mathcal {K}} ( - R)/ {\mathcal {K}} (R)) + \sum _{i \in \pi } R(i) \varepsilon _i = \mathcal {E} (R)+ \sum _{i \in \pi } R(i) \varepsilon _i. $$

We recall that $\mathcal {E}$ is the vector of the energies of the reactions in the network and is defined as in (2.8) w.r.t. $(\Omega , \mathcal {R}, \mathcal {K} )$. Hence (2.8) implies that

$$ \overline{\mathcal {E} }(R) = \sum _{i \in \pi } R(i)E(i)+ \sum _{i \in \pi } R(i) \varepsilon _i $$

Therefore $\overline{E} = E+ \varepsilon$ is an energy of the perturbed kinetic system that, as a consequence, satisfies the detailed balance condition.

The perturbed constant $c_{L,p} (\overline{\mathcal {K}}, \overline{E})$ is given by

$$\begin{aligned} c_{L,p} (\overline{\mathcal {K}}, \overline{E})&=\sum _{j\in X_L} \sum _{r \in Y_L} e^{\sum _{k=0}^{L} \overline{E}(j_k) } \prod _{i=1}^L e^{\sum _{k \in I(R_i)} \overline{E}(k) R_i(k)} \overline{K}_{R_i} R_i(j_{i-1}) R_i(j_i) \\&= \sum _{r \in Y_L}\prod _{\ell =1}^L e^{\sum _{k \in I(R_\ell )} \overline{E}(k) R_\ell (k)} \sum _{j\in X_L} e^{\sum _{k=0}^{L} \overline{E}(j_k) } \prod _{i=1}^L \overline{K}_{R_i} R_i(j_{i-1}) R_i(j_i). \end{aligned}$$

We differentiate $c_{L,p} (\overline{\mathcal {K}}, \overline{E})$ with respect to the perturbations $\delta _{\overline{R}_k}$. We recall that we perturb on the reactions that define a path connecting (1) with (*p*) that has minimal length. Then we obtain that

$$\begin{aligned} \left( \prod _{k=1}^L \frac{d}{d \delta _{\overline{R}_k} } \right) c_{L,p} (\overline{\mathcal {K}}, \overline{E}) =&\sum _{r \in Y_L}\prod _{\ell =1}^L e^{\sum _{k \in I(R_\ell )} \overline{E}(k) R_\ell (k)} \sum _{j\in X_L} e^{\sum _{k=0}^{L} \overline{E}(j_k) }\prod _{i=1}^L R_i(j_{i-1}) R_i(j_i) \cdot \\&\cdot \left( \prod _{k=1}^L \frac{d}{d \delta _{\overline{R}_k} } \right) \prod _{i=1}^L \overline{K}_{R_i} \end{aligned}$$

Notice that

$$ \left( \prod _{k=1}^L \frac{d}{d \delta _{\overline{R}_k} } \right) \prod _{i=1}^L \overline{K}_{R_i} \ne 0 \iff R_i=\overline{R}_ i \ \forall i \in \{ 1, \dots , L\}. $$

As a consequence we deduce that

$$\begin{aligned} \left( \prod _{k=1}^L \frac{d}{d \delta _{\overline{R}_k} } \right) c_{L,p} (\overline{\mathcal {K}}, \overline{E})&= \prod _{\ell =1}^L e^{\sum _{k \in I(\overline{R}_\ell )} \overline{E}(k) \overline{R}_\ell (k)} \sum _{j\in X_L} e^{\sum _{k=0}^{L} \overline{E}(j_k) }\prod _{i=1}^L \overline{R}_i(j_{i-1}) \overline{R}_i(j_i). \end{aligned}$$

We use the notation

$$\begin{aligned} \Delta _\delta c_{L,p} (\overline{\mathcal {K} }, \overline{E}) := \sum _{j\in X_L} e^{\sum _{k=0}^{L} \overline{E}(j_k) } \prod _{i=1}^L R_i(j_{i-1}) R_i(j_i). \end{aligned}$$

Notice that if $\Delta _\delta c_{L,p} (\overline{\mathcal {K} }, \overline{E} )\ne 0$. Then the proof is finished. Indeed, this would imply that

$$ \left( \prod _{k=1}^L \frac{d}{d \delta _{\overline{R}_k} } \right) c_{L,p} (\overline{\mathcal {K}}, \overline{E}) \ne 0 $$

for sufficiently small values of $\delta$. Since for $\delta =0$ we have that $c_{L,p} (\overline{\mathcal {K} }, \overline{E} ) =c_{L,p} (\mathcal {K}, E ) =0$ we have that for sufficiently small $\delta$ it holds that $c_{L,p} (\overline{\mathcal {K} }, \overline{E} )\ne 0$.

Therefore we assume that $\Delta _\delta c_{L,p} (\overline{\mathcal {K} }, \overline{E} )= 0$. We now differentiate $\Delta _\delta c_{L,p} (\overline{\mathcal {K} }, \overline{E} )$ with respect to the energy changes along the vertices of the path $\pi$, i.e. we consider

$$\begin{aligned} \left( \prod _{i \in \pi } \frac{d}{d \varepsilon _i} \right) \Delta _\delta c_{L,p} (\overline{\mathcal {K}}, \overline{E})&= \sum _{j\in X_L} \left( \prod _{i \in \pi } \frac{d}{d \varepsilon _i} \right) e^{\sum _{k=0}^{L} \overline{E}(j_k) } \prod _{i=1}^L \overline{R}_i(j_{i-1}) \overline{R}_i(j_i)\\&= e^{\sum _{k=0}^{L} \overline{E}(j^\pi _k) } \prod _{i=1}^L \overline{R}_i(j^\pi _{i-1}) \overline{R}_i(j^\pi _i) \ne 0 \end{aligned}$$

where $j^\pi _i$ are the vertices in the path $\pi$.

As a consequence we deduce that for sufficiently small values of $\varepsilon >0$ we have that $\Delta _\delta c_{L,p} ( \overline{\mathcal {K}}, \overline{E} ) \ne 0$. Hence the desired conclusion follows, i.e. we have that for sufficiently small values of $\varepsilon$ and of $\delta$ we have that $c_{L,p} ( \overline{\mathcal {K}}, \overline{E} ) \ne 0$.

In particular we have proven that there exists a rate function $\overline{\mathcal {K}}$ that satisfies the assumption of the Theorem and that is such that

$$ \varphi _{L, p} (t) = \frac{1}{(L+1)!}c_{L+1, p}(\overline{\mathcal {K}}, \overline{E}) t^{L+1} + o(t^{L+1}), \ \text { as } t \rightarrow 0^+ $$

where $$c_{L+1, p}(\overline{\mathcal {K}}, \overline{E}) \ne 0$$ and where $$\varphi $$ is the solution of (3.3) corresponding to the kinetic system $$(\Omega , \mathcal {R}, \overline{\mathcal {K} })$$. As a consequence we deduce that for every $$T>0 $$ we have $$\sup _{t \in (0,T]} |\varphi _p(t) |>0$$. Hence the solution of the system of ODEs (2.12) induced by $$(\Omega , \mathcal {R}, \overline{\mathcal {K} }) $$ is such that the property 3 in Definition 2.13 holds, i.e. $$ \sup _{ t>0} | n_p (t ) - N(p) | > 0$$. $$\square $$

### Robust adaptation in non-conservative systems that satisfy the detailed balance property

In this section we study whether a signalling system with the detailed balance property (or, more precisely, the underlying kinetic system satisfies the detailed balance property) can have the adaptation property in a stable manner, i.e. when the parameters are not fine tuned. We have several results in this direction. First of all we prove that every system that satisfies the detailed balance property, is connected, is non conservative and is such that the signal appears in one of the conservation laws, satisfies the adaptation property, unless the parameters are fine tuned.

As a second result we prove that if a signalling system is such that the underlying kinetic systems is closed and its conservation laws satisfy a suitable non-degeneracy assumption, then it does not have the adaptation property in a stable manner. The non-degeneracy assumption that we have to make on the conservation laws is a rather natural assumption. In particular, we assume that every conservation law can be written as the sum of conservation laws that contain both the product and the signal, i.e. $\forall m \in \mathcal {B}$ we have $m(1)\ne 0$ and $m(p) \ne 0$, where (1) is the signal and (*p*) is the product. We will discuss this assumption in detail in Section 3.3.

#### Proposition 3.5

Assume that the kinetic system $(\Omega , \mathcal {R}, \mathcal {K})$ satisfies the detailed balance property, assume that the chemical network $(\Omega , \mathcal {R} )$ is connected and is such that $\mathcal {M} \ne \{ 0\}$, without loss of generality we assume that there exists a $m \in \mathcal {M}$ such that $m(1)>0$. Moreover assume that the chemical network $(\Omega , \mathcal {R}, \mathcal {K})$ is not conservative. Assume that the function $n_1=f$ is an admissible signal and that the system of ODEs (2.12) admits only positive steady states. Then we have that either

- there exists a $p \in \Omega \setminus \{ 1\}$ such that the network $(\Omega , \mathcal {R}, \mathcal {K}, n_1 (t) )$ satisfies the adaptation property with respect to (*p*). Moreover, in this case there exists an open neighborhood *U* of $\mathcal {K}$, within the class of chemical rates for which the detailed balance property is satisfied, such that the property of adaptation holds for every $\overline{\mathcal {K}}\in U$;
- or alternatively, we have that for every $\delta >0$ there exists a rate function $\overline{\mathcal {K}}: \mathcal {R} \rightarrow \mathbb {R}_+$ satisfying the assumptions of Theorem 3.3 and such that $(\Omega , \mathcal {R}, \overline{\mathcal {K}}, n_1(t) )$ satisfies the adaptation property.

#### Proof

Let $\overline{n}_1$ be such that $\lim _{t \rightarrow \infty } f(t)= \overline{n}_1$. Then Proposition 2.15 implies that there exists an energy $\overline{E}$ of the kinetic system $(\Omega , \mathcal {R}, \mathcal {K} )$ such that $\lim _{ t \rightarrow \infty } n (t) = e^{- \overline{E}}$. This in particular implies that the property 1 in the Definition 2.13 holds.

Since the chemical network $$(\Omega , \mathcal {R})$$ is non-conservative there exists a $$p \in \Omega \setminus \{1\} $$ that is such that $$m (p )=0$$ for every $$m \in \mathcal {M}$$. As a consequence we deduce that all the energies *E* of the kinetic system $$(\Omega , \mathcal {R}, \mathcal {K} ) $$ are such that $$E(p)=\overline{E}$$. Indeed, assume by contradiction that there exists two energies $$E_1$$ and $$E_2 $$ satisfying (2.8) and that are such that $$E_1(p) \ne E_2(p)$$. This implies that $${\textbf {R}}^T (E_1- E_2 ) =0$$, hence that $$E_1- E_2 \in \mathcal {M} $$. Since the kinetic system is non conservative in (*p*) we have that every $$m \in \mathcal {M} $$ is such that $$m (p) =0 $$. This implies that $$E_1(p)=E_2(p)$$, a contradiction. As a consequence we have that every steady state of $$(\Omega , \mathcal {R}, \mathcal {K} ) $$ is such that $$N (p)=e^{- \overline{E}(p)} $$. Hence property 2. in the Definition of adaptation holds.

Finally since the assumptions of Theorem 3.3 hold, also the third property necessary to have the adaptation property holds. $\square$

#### Remark 3.6

The meaning of point (b) in Proposition 3.5 is that even if the properties 1 and 2 in the definition of adaptation (Definition 2.13) hold, under the assumptions of Proposition 3.5, the adaptation property could fail due the absence of response to a signal for a fine-tuned choice of the chemical rates (see Example 3.2 ). Point (b) in Proposition 3.5 states that such absence of response is unstable under small perturbation of the chemical rates.

#### Remark 3.7

Proposition 3.5 gives a simple mechanism to construct bidirectional kinetic systems that satisfy the property of adaptation in a stable manner.

#### Example 3.8

(*Non conservative signalling system with detailed balance and with the adaptation property*) Consider the chemical reactions

$$ (1) \overset{K_1 }{\underset{K_1 e^{ E_2 + E_3- E_1 }}{\leftrightarrows }} (2) +(3), \quad (3) \overset{K_2 }{\underset{K_2 e^{ E_1 -E_3 }}{\leftrightarrows }} (1). $$

This kinetic system is not conservative as we have that $m (3)=0$ for every $m \in \mathcal {M}$. The system does not have cycles, hence the detailed balance property holds. Moreover since the network is connected we have that a change in the concentration of (1) produces a change in (3) , unless the parameters are fine tuned (see Theorem 3.3). The steady states $N \in \mathbb {R}_+^3$ of the system of ODEs (2.4) associated with the kinetic system have the following form

$$ N_i = e^{- \mathcal {E}(i) } $$

where $$ \mathcal {E} =( \mathcal {E}(i))_{i=1}^3 \in \mathbb {R}^3 $$ is given by $$E= (E_1, E_2, E_3 ) + m $$ for some $$m \in \mathcal {M}$$. Since we have that $$m (3)=0$$ for every $$m \in \mathcal {M} $$ we have that $$N_3 = e^{-E_3} $$ and is independent on the concentration of the signal (1). Therefore the adaptation property holds and is stable under perturbation of the chemical rates that preserve the property of detailed balance.

#### Fluxes of chemicals required in order to have adaptation with detailed balance

Proposition 3.5 guarantees that the property of robust adaptation is achieved by signalling systems that satisfy the property of detailed balance and that are not conservative. In this section we explain how to complete these signalling systems in order to write them as thermodynamically admissible systems of the form (1.3). In this section we restrict the attention to kinetic systems with detailed balance that exchange only one substance (different from the signal) with the environment, i.e. we assume that there exists a unique substance $p \in \Omega$ such that $m (p)=0$ for every $m \in \mathcal {M}$. The same arguments can be applied to situations in which there are several substances $\{p_j \}_{j=1}^k$ such that $m(p_j)=0$ for every $m \in \mathcal {M}$.

##### Proposition 3.9

Assume that $$(\Omega , \mathcal {R}, \mathcal {K}, n_1(t)) $$ is a signalling system of the form (2.12) and let *f* be an admissible signal. Assume that the kinetic system $$(\Omega , \mathcal {R}, \mathcal {K}) $$ satisfies the detailed balance property and that there exists a unique $$p \in \Omega $$ with $$p \ne 1 $$ such that $$m (p)=0, \ \forall m \in \mathcal {M} $$. Then there exists a kinetic system $$(\Omega _c, \mathcal {R}_c, \mathcal {K}_c) $$ with $$ \Omega _c = \Omega \cup \{ N+1\}$$ that satisfies the property of detailed balance, it is conservative and it is such that the following property holds. Consider the fluxes $$J^c_R $$ given by (2.6) associated to the kinetic system $$(\Omega _c, \mathcal {R}_c, \mathcal {K}_c)$$. Then the solution $$n=(n_1, n_2, \dots , n_{N+1} ) \in \mathbb {R}^{N+1} $$ to the equation

3.10 $$\begin{aligned} \frac{d n (t) }{dt} = \sum _{R \in \mathcal {R}_c } J^c_R(n) R + J^{ext} (t), \quad n(0) \in \mathbb {R}_+^{N+2} \end{aligned}$$

where

3.11 $$\begin{aligned} J^{ext}(t) = - \sum _{(\mathcal {R}_c)_s} e_{N+1} + \left( J^c_R(n) R (p)+ \frac{ d f(t) }{dt } - \sum _{R \in \mathcal {R}_c } J^c_R(n) R(1) \right) e_{1} \end{aligned}$$

is such that the vector $(n_1, n_2, \dots , n_N ) \in \mathbb {R}^{N}$ is the solution to the system (2.12) corresponding to the signalling system $(\Omega , \mathcal {R}, \mathcal {K}, n_1(t))$ with $n_1(t)=f(t)$ and initial condition $(n_1(0), n_2(0), \dots , n_N(0))$.

##### Proof

First of all we construct a completion of $(\Omega , \mathcal {K}, \mathcal {R})$. Let $\Omega _c: = \{ 1, \dots , N, N+1 \}$ and let

$$ \mathcal {R}_c := \{ (R, -R(p) )^T \in \mathbb {Z}^{N+1} : R \in \mathcal {R} \}. $$

We define the rate function $\mathcal {K}_c: \mathcal {R} \rightarrow \mathbb {R}_+$ as

3.12 $$\begin{aligned} \mathcal {K}_c(R)={\left\{ \begin{array}{ll} \frac{K_R}{ n_{N+1}(0) } & {\textit{ if }} p \in I(R) \\ K_R & \textit{ if } p \in I(R). \end{array}\right. } \end{aligned}$$

Notice that $(\Omega _c, \mathcal {R}_c, \mathcal {K}_c)$ is conservative, indeed $(0,\dots , 0, 1, 1 ) \in \mathbb {R}^{N+1} \in \mathcal {M}_c= \operatorname {span} \{ R: R \in \mathcal {R}_c \}^{\perp }$ and due to the fact that *p* is the unique element of $\Omega$ such that $m(p)=0$ for every $m \in \mathcal {M}$. We now prove that the kinetic system, $(\Omega _c, \mathcal {R}_c, \mathcal {K}_c)$ satisfies the property of detailed balance. By construction, the cycles of the completed system $(\Omega _c, \mathcal {R}_c, \mathcal {K}_c )$ coincide with the cycles of the system $(\Omega , \mathcal {R}, \mathcal {K} )$. Moreover we have that for every cycle $c \in \mathcal {C} = \mathcal {C}_c$ it holds that

$$\begin{aligned} \prod _{ R_j \in {(\mathcal {R}_c)}_s } \left( \frac{{\mathcal {K}}_c(R_j) }{{\mathcal {K}}_c(-R_j)} \right) ^{c(j)}&= \prod _{ \{ R_j \in \mathcal {R}_s : R_j(p)=0 \}} \left( \frac{K_{R_j}}{K_{-R_j}} \right) ^{c(j)} \prod _{ \{ R_j \in \mathcal {R}_s : p \in I(R_j) \} } \left( \frac{K_{R_j} n_{N+1}(0) }{K_{-R_j}} \right) ^{c(j)} \cdot \\&\cdot \prod _{ \{ R_j \in \mathcal {R}_s : p \in F(R_j) \} } \left( \frac{K_{R_j}}{K_{-R_j} n_{N+1}(0)} \right) ^{c(j)} \\&= \prod _{ R_j \in \mathcal {R}_s } \left( \frac{K_{R_j}}{K_{-R_j}} \right) ^{c(j)} \left( \frac{ \prod _{ R_j \in \mathcal {R}_s : p \in I(R_j)} n_{N+1}(0) }{\prod _{ R_j \in \mathcal {R}_s: p \in I(-R_j) } n_{N+1}(0) } \right) ^{c(j)} \\&= \prod _{ R_j \in \mathcal {R}_s } \left( \frac{K_{R_j}}{K_{-R_j}} \right) ^{c(j)} =1. \end{aligned}$$

For the last inequality we used the Wegscheider criterion (Lemma 2.11) and the fact that $$(\Omega , \mathcal {K}, \mathcal {R}) $$ satisfies the detailed balance property. Using the Wegscheider criterion (Lemma 2.11) again we deduce that the completed system $$(\Omega _c, \mathcal {R}_c, \mathcal {K}_c) $$ satisfies the detailed balance property. Then $$(\Omega _c, \mathcal {R}_c, \mathcal {K}_c)$$ is closed. In order to conclude our proof notice $$J^{ext} $$ in (3.10)-(3.11) is such that $$n_{N+1} (t) = n_{N+1}(0)$$ for every $$t >0 $$. Moreover the choice of chemical rates (3.12) guarantees that

$$ J^c_R ( (n_1, n_2, \dots , n_{N+1} (0) )^T) = J_{\overline{R}} ( (n_1, n_2, \dots , n_{N} )^T) $$

for every *R* such that $R=(\overline{R}, - \overline{R}(p))^T \in \mathcal {R}_c$. As a consequence the system (3.10) can be rewritten as

$$\begin{aligned} n_1(t)&=f(t) \\ \frac{d n_i (t) }{dt}&= \sum _{R \in \mathcal {R}_c } J^c_R(n) R(i) = \sum _{R \in \mathcal {R} } J_R(n) R(i) , \quad i \in \Omega \setminus \{ 1 \} , \\ n_{N+1} (t)&= n_{N+1} (0) \end{aligned}$$

and the desired conclusion follows. $\square$

Proposition 3.9 provides a thermodynamically admissible signalling system whose dynamics is equivalent to (2.12).

### Robust adaptation is impossible in closed signalling systems

In this section we prove that if the conservation laws of a kinetic system satisfy a suitable non-degeneracy property, then the fact that the kinetic system satisfies the adaptation property implies that the kinetic system is not closed. We start the section by explaining the property that the conservation laws must satisfy.

First of all let us consider the basis of the extreme rays $\mathcal {B} = \{ m_i \}_{i=1}^L \subset \mathcal {M}_+$ of $\mathcal {M}$. Consider $i \in \Omega$ and let us define the vector $m^{(i)} \in \mathbb {R}_*^L$ that is such that

$$ m^{(i)}(j) := m_j(i). $$

#### Definition 3.10

($\mathcal {M}$-*connectivity*) Let $(\Omega , \mathcal {R}, \mathcal {K}, n_1(t) )$ be a signalling system and let $\mathcal {M}$ be the associated set of conservation laws. We say the two elements $i, j \in \Omega$ are $\mathcal {M}$-connected if $\langle m^{(i)}, m^{(j)}\rangle \ne 0$. We say that the signalling system $(\Omega , \mathcal {K} )$ is $\mathcal {M}$-connected if every $i, j \in \Omega$ are $\mathcal {M}$-connected.

When there exist two elements $i, j \in \Omega$ such that $\langle m^{(i)}, m^{(j)} \rangle \ne 0$ we say that the system satisfies a *factorization assumption*.

#### Example 3.11

(*Adaptation property when* $\mathcal {M}$-*connectivity fails*) Consider the chemical system induced by the following reactions

$$ (1) + (3) \leftrightarrows (2) + (4),\quad (1) \leftrightarrows (2), \quad (3) \leftrightarrows (4). $$

The basis of extreme rays $$\mathcal {B} $$ is given by $$\mathcal {B} =\{ (1,1,0,0)^T, (0,0,1,1)^T \} $$. In particular we have that $$m^{(1)} =(1,0)^T $$ and that $$m^{(4)} =(0,1)^T $$, therefore $$ \langle m^{(1)}, m^{(4)} \rangle = 0 $$. Hence the substance (1) and the substance (4) are not $$\mathcal {M} $$ connected. Moreover notice that the chemical network is conservative. We can associate to the network a rate function that satisfies the detailed balance condition. We can assume that $$n_1=f$$ is an admissible signal. Then by Proposition 2.15 we know that the system converges to a steady state *N* that satisfies (2.15), hence in particular we have that

$$ N_3 + N_4 - n_3(0) - n_4(0)=0 \ \text { and } \ \frac{N_3}{N_4}=\frac{n_3(0)}{n_4(0)}. $$

This implies that $$N_4 $$ does not depend on the signal $$n_1=f$$. Since we have that Theorem 3.3 guarantees that property 2. in the Definition of adaptation holds we deduce that this kinetic system satisfies the adaptation property even if it is closed.

#### Theorem 3.12

Let $(\Omega ,\mathcal {R}, \mathcal {K}, n_s )$ be a signalling kinetic system is $\mathcal {M}$-connected. Assume that the adaptation property holds, then

- either the kinetic system $(\Omega ,\mathcal {R}, \mathcal {K} )$ is not closed,
- or for every $\delta >0$ there exists a map $\mathcal {K}_\delta : \mathcal {R} \rightarrow \mathbb {R}_+$ such that
  $$ \Vert \mathcal {K}- \mathcal {K}_\delta \Vert < \delta $$
  and such that $(\Omega , \mathcal {R}, \mathcal {K}_\delta )$ does not satisfy the adaptation property.

#### Proof

Let us assume that $(\Omega ,\mathcal {R}, \mathcal {K} )$ is closed, hence it satisfies the detailed balance property. Proposition 2.15 together with the property 1 of Definition 2.13 implies that there exists an energy *E* of $(\Omega , \mathcal {R}, \mathcal {K} )$ that is such that $n(t) \rightarrow e^{- E }$ as $t \rightarrow \infty$. Let $\mathcal {B} = \{ m_k\}_{k=1}^L$. Using (2.15) we deduce that for every $k \in \{ 1, \dots , L \}$ it holds that

$$ m_k^T e^{-E } - m_k^T e^{- E_0 } =m^{(1)}_k \overline{J}_1^F $$

where $$n_0=e^{- E_0} $$. Since *E* and $$E_0 $$ are two energies of the same kinetic system we have that $$E=E_0 + \sum _{i =1}^L m_i \eta _i $$ for some chemical potentials $$\{ \eta _i \}_{i=1}^L $$ with $$\eta _i \in \mathbb {R}$$. Hence the above equality reduces to

$$ m^{(1)}_k \overline{J}_1^F = \sum _{j \in \Omega } m_k^{(j)} e^{- E_0 (j) } \left( e^{\sum _{i =1}^L m^{(j)}_i \eta _i} -1 \right) . $$

Let us stress that the chemical potentials $\eta _i$ depend on $\overline{J}_1^F$. Therefore differentiating the above equality with respect to $\overline{J}_1^F$ we deduce that the property 2 in Definition 2.13 implies that

$$ m^{(1)}_k = \sum _{j \in \Omega } m_k^{(j)} e^{- E (j) } \sum _{i=1}^L m_i^{(j)} \frac{\partial \eta _i}{\partial _{\overline{J}_1^F}}. $$

This equality can be rewritten as

$$ m^{(1)}= M F(E) M^T \xi $$

where $M \in \mathbb {R}^{L \times N }$ with $M_{ij}=m^{(j)}_i$, $F(E)=\operatorname {diag} \{ e^{-E(i)} \}_{i=1}^N$ and where $\xi \in \mathbb {R}^L$ is defined as $\xi (i)=\frac{\partial \eta _i}{\partial _{\overline{J}_1^F}}$. On the other hand, since the adaptation property holds, we have that

$$ m_k^{(N)} e^{-E(N) } - m_k^{(N)} e^{- E_0(N)} =0 $$

where $N=p$ is the product. As a consequence, differentiating with respect to $\overline{J}_1^F$ also this equation, we deduce that the adaptation property implies that

3.13 $$\begin{aligned} {\left\{ \begin{array}{ll} M F(E) M^T \xi & = m^{(1)}\\ \langle m^{(N)}, \xi \rangle & =0. \end{array}\right. } \end{aligned}$$

Let us define the matrix $\mathcal {D} (\zeta ) \in \mathbb {R}^{N \times N}$ as

$$ \mathcal {D} (\zeta ):= \sum _{j=1}^N \zeta _j \left( m^{(j)} \otimes m^{(j)} \right) = M F(E) M^T, $$

where we use the notation $\zeta = e^{- E }$.

Since the kinetic system $(\Omega , \mathcal {R}, \mathcal {K} )$ is conservative, the matrix $\mathcal {D}(\zeta )$ is positive definite by definition, hence we can diagonalize it, i.e.

$$ \mathcal {D} (\zeta )= \sum _{j=1}^N \lambda _j (\zeta ) \left( \eta _j (\zeta ) \otimes \eta _j (\zeta ) \right) $$

where $\{ \eta _j(\zeta ) \}$ is an orthonormal basis of $\mathbb {R}^N$ and $\{ \lambda _j (\zeta ) \}$ are the eigenvalues of $\mathcal {D}(\zeta )$. Notice that since $\mathcal {D}(\zeta )$ is positive definite we have that for every $j \in \{ 1, \dots , N\}$ we have that $\lambda _j (\zeta ) >0$. Moreover, the inverse of $\mathcal {D} (\zeta )$ is given by

$$ \mathcal {D} (\zeta )^{-1} = \sum _{j=1}^N \frac{1}{\lambda _j (\zeta )} \left( \eta _j (\zeta ) \otimes \eta _j (\zeta ) \right) . $$

This, together with (3.13) implies that $\xi = \mathcal {D} (\zeta )^{-1}m^{(1)}$.

Using the fact that $\langle m^{(N)}, \xi \rangle =0$ and that the matrix $\mathcal {D}(\zeta )^{-1}$ is symmetric we deduce that the property of adaptation implies that

$$ \langle m^{(N)}, \mathcal {D} (\zeta )^{-1}m^{(1)} \rangle = \langle m^{(1)}, \mathcal {D} (\zeta )^{-1} m^{(N)} \rangle =0 . $$

We define the following equivalence relation. We say that $i \sim j$ if and only if we have that either

$$ \langle m^{(i)} ,\mathcal {D} (\zeta )^{-1} m^{(k)} \rangle \ne 0 $$

or, alternatively for every $\delta >0$ there exists a $x \in B_\delta (\zeta ) \subset \mathbb {R}^L$ such that

$$ \langle m^{(i)} ,\mathcal {D} (x)^{-1} m^{(k)} \rangle \ne 0, \quad \forall z \in B_\delta (\zeta ). $$

The relation $\sim$ is an equivalence relation. Indeed, we have that for every $i \in \Omega$ it holds that $(i) \sim (i)$, because $\mathcal {D}(\zeta )$ is positive definite. Moreover the symmetry of $\mathcal {D}^{-1}(\zeta )$ implies that $(i) \sim (j)$ iff $(j) \sim (i)$. Finally we prove the transitive property. We have to prove that the fact that $(i) \sim (j)$ and $(j) \sim (k)$ implies $(i) \sim (k)$. Indeed, notice that by the definition of $\mathcal {D}(\zeta )$ we have

$$ \frac{\partial }{\partial _{\zeta _j} }\mathcal {D} (\zeta )^{-1} = - \mathcal {D} (\zeta )^{-1} \left( m^{(j)} \otimes m^{(j)} \right) \mathcal {D} (\zeta )^{-1}. $$

Therefore, if we assume that $\langle m^{(i)}, \mathcal {D} (\zeta )^{-1} m^{(j)} \rangle \langle m^{(j)}, \mathcal {D} (\zeta )^{-1} m^{(k)} \rangle \ne 0$, then

$$\begin{aligned} \frac{\partial }{\partial _{\zeta _j} } \langle m^{(i)} ,\mathcal {D} (\zeta )^{-1} m^{(k)} \rangle&= \langle m^{(i)} , \frac{\partial }{\partial _{\zeta _j} } \mathcal {D} (\zeta )^{-1} m^{(k)} \rangle = - \langle m^{(i)} , \mathcal {D} (\zeta )^{-1} \left( m^{(j)} \otimes m^{(j)} \right) \\&\quad \mathcal {D} (\zeta )^{-1} m^{(k)} \rangle \\&= - \langle m^{(i)} , \mathcal {D} (\zeta )^{-1} m^{(j)} \rangle \langle m^{(j)} , \mathcal {D} (\zeta )^{-1} m^{(k)} \rangle \ne 0. \end{aligned}$$

This in particular implies that for every $\delta >0$ there exists a $z \in B_\delta (\zeta )$ such that $\langle m^{(i)},\mathcal {D} (z)^{-1} m^{(k)} \rangle \ne 0$. Hence $(i) \sim (k)$. Assume instead that $\langle m^{(i)}, \mathcal {D} (\zeta )^{-1} m^{(j)} \rangle \langle m^{(j)}, \mathcal {D} (\zeta )^{-1} m^{(k)} \rangle =0$. Since $(i) \sim (j)$ and $(j) \sim (k)$ we know that for every $\delta >0$ there exists a $z \in B_\delta (\zeta )$ such that

$$ \langle m^{(i)} , \mathcal {D} (z)^{-1} m^{(j)} \rangle \langle m^{(j)} , \mathcal {D} (z)^{-1} m^{(k)} \rangle \ne 0. $$

The above computation shows that for every $\delta >0$ there exists a $x \in B_\delta (z)$ such that $\langle \mathcal {D} (x)^{-1} m^{(k)} \rangle \ne 0$. Notice that this implies that $(i) \sim (k)$.

We denote with $\mathbb {M}_1$ the equivalence class (corresponding to the equivalence relation $\sim$) that contains $m^{(1)}$. We want to prove that the fact that the kinetic system is $\mathcal {M}$-connected implies that, upon small perturbation of the reaction rates, we have $\mathbb {M}_1=\{ m^{(i)} \}_{i \in \Omega }$. To this end we prove that if there exist more than one equivalence class induced by $\sim$, then the kinetic system $(\Omega , \mathcal {R}, \mathcal {K} )$ is not $\mathcal {M}$-connected. So let us assume that there exists at least two equivalence classes on the set $\{ m^{(i)} \}_{i \in \Omega }$ induced by $\sim$. We define the following two sets

$$ W_1 := \{ y \in \mathbb {R}^L :\langle y, \mathcal {D}(\zeta )^{-1} m^{(k)}\rangle =0, \quad \forall m^{(k)} \in \mathbb {M}_1 \} $$

and

$$ W_2:= \{ y \in \mathbb {R}^L :\langle y, \mathcal {D}(\zeta )^{-1} m^{(k)}\rangle =0, \quad \forall m^{(k)} \in \{ m^{(i)} \}_{i \in \Omega } \setminus \mathbb {M}_1 \}. $$

We now want to prove that $W_1 \cap W_2 = \{ 0\}$. To this end recall that, since the rank of the matrix *M* is *L*, we have that there exists a subset $\{ m^{(i_j)} \}_{j=1 }^L$ of $\{ m^{(i)} \}_{i \in \Omega }$ of linearly independent vectors. As a consequence the corresponding vectors $\{ \xi ^{(i_j)} \}_{j=1 }^L$ with $\xi ^{(i_j)}:= \mathcal {D}(\zeta )^{-1} m^{(i_j)}$ are also linearly independent. Indeed assume that $\{ \alpha _{j}\}_{j =1}^L$ is such that

$$ 0=\sum _{j =1}^L \alpha _j \xi ^{(i_j)}= \mathcal {D}(\zeta )^{-1} \sum _{j =1}^L \alpha _j m^{(i_j)} $$

Since $\mathcal {D}(\zeta )^{-1}$ is invertible this implies that $\sum _{j =1}^L \alpha _j m^{(i_j)}=0$. Hence $\alpha _j=0$ for every $j \in \{ 1, \dots , L \}$. As a consequence we deduce that if $y \in W_1 \cap W_2 = \{ 0\}$ then $\langle y, \xi ^{(i_j)} \rangle =0$ for every $j \in \{ 1, \dots , L \}$. Hence $y=0$.

We prove that $W_1 \oplus W_2 = \mathbb {R}^L$. Without loss of generality assume that the number of vectors of the basis $\{ \xi ^{(i_j)} \}_{j=1}^L$ contained in the class $\mathbb {M}_1$ is given by $\gamma _1$. As a consequence we have that $\dim W_1 = L- \gamma _1$ while the dimension of $W_2 = \gamma _1$. As a consequence, since $W_1 \cap W_2 = \{ 0\}$ we deduce that $\dim (W_1+W_2)=L$, hence the desired conclusion follows and we obtain that $W_1 \oplus W_2 = \mathbb {R}^L$.

Let us define $\Omega _1 =\{ k: m^{(k)} \in W_1 \}$ and $\Omega _2 =\{ k: m^{(k)} \in W_2 \}$. Then by the definition of conservation law we have that

$$ 0 = \sum _{j \in \Omega } R(j) m^{(j) } = \sum _{j \in \Omega _1 } \pi _{\Omega _1} R(j) m^{(j) } + \sum _{j \in \Omega _2 } \pi _{\Omega _2} R(j) m^{(j) }. $$

Here we are using the notation $\pi _A v \in \mathbb {R}^{|A|}$

$$ \pi _A v :=\left( v(i)\right) _{i \in A}=(v( \min (A)), \dots , v(\max A)), $$

where $A \subset \Omega$. This implies that

$$ \sum _{j \in \Omega _1 } \pi _{\Omega _1} R(j) m^{(j) } =- \sum _{j \in \Omega _2 } \pi _{\Omega _2} R(j) m^{(j) } \in W_1 \cap W_2 = \{ 0\} . $$

This implies that every vector $m_k$ that belongs to the extremal basis $\mathcal {B}$ can be written as the sum of two conservation laws, i.e. $m_k=(\pi _{\Omega _1} m_k, 0 ) + (\pi _{\Omega _2} m_k, 0 )$. However, since $\mathcal {B}$ is a basis of extreme rays of $\mathcal {M}_+$ we have that this implies either that $\pi _{\Omega _1} m_k=0$ or that $\pi _{\Omega _2} m_k=0$. Hence this implies that $(\Omega , \mathcal {R}, \mathcal {K} )$ is not $\mathcal {M}$-connected. This concludes the proof of the fact that $\mathcal {M}$-connectivity implies that there exists a unique equivalence class induced by $\sim$.

In particular this implies that either

$$ \langle \mathcal {D}(\zeta ) m^{(1)}, m^{(N) } \rangle \ne 0 $$

or alternatively, if $\langle \mathcal {D}(\zeta ) m^{(1)}, m^{(N) } \rangle = 0$, then for every $\delta >0$ there exists a $z\in B_\delta (\zeta )$ such that

$$ \langle \mathcal {D}(z) m^{(1)}, m^{(N) } \rangle \ne 0. $$

This implies the statement of the theorem. Indeed recall that $\zeta = e^{-E}$. Hence for any $\delta >0$ we can construct the rate function $\mathcal {K}_\delta : \mathcal {R} \rightarrow \mathbb {R}_*$ in such a way that

$$ \frac{\mathcal {K}_\delta (-R) }{\mathcal {K}_\delta (R) }= e^{- \sum _{i \in \Omega } \overline{E}(i) R(i) } \quad \forall R \in \mathcal {R}_s. $$

where $z=e^{- \overline{E}}$. Then the kinetic system $(\Omega , \mathcal {R}, \mathcal {K}_\delta )$ does not satisfy the adaptation property, because (3.13) fails. $\square$

### An example of robust invariance of the concentration of product at steady state on the signal in signalling systems without detailed balance

In this section we present a signalling system that is conservative, does not satisfy the property of detailed balance and satisfies the property 2. in the definition of adaptation (Definition 2.13) in a stable manner. As we will see later, this model has conservation laws that are such that the signal and the product are not $\mathcal {M}$-connected.

#### Example 3.13

(*Robust invariance of some concentrations at steady state on the signal concentration in signalling systems without detailed balance*) We consider the following chemical reactions

$$ (1) + (2) \underset{K_{-1} }{\overset{K_1}{\rightleftarrows }\ } (3), \quad (1)+(4) \underset{K_{-2} }{\overset{K_2}{\rightleftarrows }\ } (3) , \quad (5)+(2) \underset{K_{-3} }{\overset{K_3}{\rightleftarrows }\ } (4) , \quad (5) \underset{K_{-4} }{\overset{K_4}{\rightleftarrows }\ } (6) \underset{K_{-5} }{\overset{K_5}{\rightleftarrows }\ } (7) \underset{K_{-6} }{\overset{K_6}{\rightleftarrows }\ } (5). $$

The set of the reactions $\mathcal {R}_s$ is given by $\mathcal {R}_s := \left\{ R_i \right\} _{i=1}^{6}$ where

3.14 $$\begin{aligned}&R_1 =(-1,-1,1,0,0,0,0)^T, R_2=(-1,0,1,-1,0,0,0)^T, R_3=(0,-1,0,1,-1,0,0)^T,\\&R_4=(0,0,0,0,-1,1,0)^T, R_5=(0,0,0,0,0,-1,1)^T, R_6=(0,0,0,0,1,0,-1)^T. \nonumber \end{aligned}$$

We assume that the evolution in time of the signal (1) is a given function of time, i.e. $$n_1(t)= f(t) $$ where *f* is an admissible signal converging to $$\overline{n}_1>0$$. Then the chemical reactions define a signalling system that is conservative. Indeed we have that the vector $$m =(1,1,2,1,1,1,1)^T$$ is a conserved quantity. Notice that the matrix of the conserved quantities *M* is given by

$$ M = \begin{bmatrix} m_1^T \\ m_2^T \\ m_3^T \end{bmatrix} = \begin{bmatrix} 0 & 1 & 1 & 1 & 0& 0& 0 \\ 1 & 0 & 1 & 0& 0& 0 & 0 \\ 0 & 0 & 0 & 0 & 1 & 1 & 1 \end{bmatrix} . $$

Notice that we have that the signal (1) is not $\mathcal {M}$-connected (as in Definition 3.10) with the substances (5), (6), (7). Then we have that

$$\begin{aligned} \frac{d n_2}{ dt }&= R_1(2) J_{R_1} (n) + R_3(2) J_{R_3} (n) \\ \frac{d n_3}{ dt }&= R_1(3) J_{R_1} (n) + R_2(3) J_{R_2} (n) \\ \frac{d n_4}{ dt }&= R_2(4) J_{R_2} (n) + R_3(4) J_{R_3} (n) \\ \frac{d n_5}{ dt }&= R_3(5) J_{R_3} (n) + R_4(5) J_{R_4} (n) + R_6(5) J_{R_6} (n) \\ \frac{d n_6}{ dt }&= R_4(6) J_{R_4} (n) + R_5(6) J_{R_5} (n) \\ \frac{d n_7}{ dt }&= R_5(7) J_{R_5} (n) + R_6(7) J_{R_6} (n). \end{aligned}$$

Using (3.14), we deduce that the first three equations evaluated at a steady state $N \in \mathbb {R}_+^7$ imply that

$$ J_{R_1} (N) = J_{R_2} (N) = J_{R_3} (N) =0. $$

In particular this implies that $$ J_{R_4} (N) = J_{R_5} (N) = J_{R_6} (N) $$. Since the substances (1), (2), (3) and (4) do not appear in the chemical reactions $$R_4 $$, $$R_5 $$ and $$R_6 $$ then the concentration of (1) , (2), (3) and (4) do not affect the fluxes $$ J_{R_4} (N) $$, $$ J_{R_5} (N) $$ and $$ J_{R_6} (N) $$. The conditions $$ J_{R_4} (N) = J_{R_5} (N) = J_{R_6} (N) $$ and $$J_{R_1} (N) = J_{R_2} (N) = J_{R_3} (N) =0$$ define a family of positive steady states parametrized by the conservation laws $$m \in \mathcal {M} $$. On the other hand recall that the steady state describing the long time behaviour must satisfy the condition (2.15) imposed by the conservation laws. Since the conserved quantities factorize the set of the substances in $$\Omega _1:=\{ 1,2,3,4 \} $$ and $$\Omega _2:= \Omega \setminus \Omega _1 $$ then we deduce that the concentration of the substances (5) (6) and (7) at steady state do not depend on the substance (1), i.e. they do not depend on $$\overline{n}_1 >0 $$. Hence the property 2. in the definition of adaptation holds and is stable upon perturbation of the chemical rates. Notice that this result does not depend on whether or not detailed balance holds. Finally we notice that this system has deficiency $$\delta $$ equal to zero (see (Feinberg 2019) for the definition of deficiency) and so the steady states are asymptotically stable.

In order to construct the example above we implicitly used the concept of buffering structure mentioned in the introduction, that has been used in order to study the sensitivity property of kinetic systems in Hirono et al. (2025); Hirono et al. (2023); Okada and Mochizuki (2016).

Given a chemical network, a buffering structure $\Gamma$ is a subnetwork (i.e. a set of substances $\Omega _\Gamma$ and a set of reactions $\mathcal {R}_\Omega$) that is such that the chemical rates of the reactions in $\Gamma$ do not influence the concentrations of the steady states of the substances of the network that are not in the buffering structure $\Gamma$. A subnetwork $\Gamma$ is a buffering structure if its influence index $\lambda (\Gamma )$ is equal to zero. The influence index $\lambda (\Gamma )$ is defined as $\lambda (\Gamma )= - |\Omega _\Gamma |$+ $|\mathcal {R}_\Gamma | - |\mathcal {C}_\Gamma |$. Here $|\mathcal {C}_\Gamma |$ is the dimension of the space of the cycles of reactions in $\mathcal {R}_\Gamma$, i.e. the dimension of the space $\{ c \in \mathbb {R}_+^{|\mathcal {R}_\Gamma |}: \sum _{k=1}^{|\mathcal {R}_\Gamma | } c(k) R_k =0 \}$. It is proven in Hirono et al. (2025); Hirono et al. (2023); Okada and Mochizuki (2016) that the rates of the chemical reactions that belong to a buffering structure $\Gamma$ do not influence the concentration at steady state of the substances that do not belong to $\Gamma$.

We explain how we can apply this result to the signalling system in Example 3.13. We can reformulate the system as an effective reduced kinetic system in which the substance (1) does not explicitly contribute to the chemical reactions, but its presence is encoded in the chemical rates. More precisely the reduced kinetic system is the following

$$ (2) \underset{K_{-1} }{\overset{K_1(f)}{\rightleftarrows }\ } (3), \quad (4) \underset{K_{-2} }{\overset{K_2(f)}{\rightleftarrows }\ } (3) , \quad (5)+(2) \underset{K_{-3} }{\overset{K_3}{\rightleftarrows }\ } (4) , \quad (5) \underset{K_{-4} }{\overset{K_4}{\rightleftarrows }\ } (6) \underset{K_{-5} }{\overset{K_5}{\rightleftarrows }\ } (7) \underset{K_{-6} }{\overset{K_6}{\rightleftarrows }\ } (5). $$

where $$K_1(f) = f(t) K_1 $$ and $$K_2(f) = f(t) K_2 $$. Let us consider the subnetwork $$\Gamma $$ with $$\Omega _\Gamma = \{2, 3, 4 \} $$ and $$\mathcal {R}_\Gamma =\{ R_1, - R_1, R_2, - R_2, R_3, - R_3 \} $$ with $$R_1= (-1,1,0, 0,0 )^T$$, $$R_2= (0,1,-1,0,0)^T$$ and $$R_3= (-1,0,1,-1,0,0)^T$$. Notice that $$\lambda (\Gamma )= 0$$ because $$|V_\Gamma |= 3 $$, $$ |\mathcal {R}_\Gamma |= 6 $$ and $$\mathcal |C_\Gamma |= 3 $$. Therefore $$\Gamma $$ is a buffering structure and the concentration at steady states of the substances (5), (6), (7) does not depend on $$K_1 (f) $$ and on $$K_2(f)$$. However we stress that this is not enough to conclude that the concentrations of the substances (5), (6) and (7) as time tends to infinity tend to a steady state that is independent on the $$\overline{n}_1 $$. Indeed, the steady state describing the long time behaviour could depend on $$\overline{n}_1$$ if (1) and (5), (6), (7) belong to the same conservation law. This is the reason why in order to obtain the adaptation property we assume that (1), (2), (3), (4) are not $$\mathcal {M} $$ connected with (5), (6), (7) , i.e. the factorization assumption holds.

Therefore, the existence of a buffering structure does not always imply that the property of robust adaptation (as defined in this paper) holds. We illustrate this using the following example in which we present a model with a buffering structure, that does not have the property 2. required to have adaptation.

#### Example 3.14

We consider the following chemical reactions

$$ (1) + (2) \underset{K_{-1} }{\overset{K_1}{\rightleftarrows }\ } (3) , \quad (3) \underset{K_{-2} }{\overset{K_2}{\rightleftarrows }\ } (4) \underset{K_{-3} }{\overset{K_3}{\rightleftarrows }\ } (5) \underset{K_{-4} }{\overset{K_4}{\rightleftarrows }\ } (3). $$

The set of the reactions $\mathcal {R}_s$ is given by $\mathcal {R}_s := \left\{ R_i \right\} _{i=1}^{4}$ where $R_1 =(-1,-1,1,0,0)^T$, $R_2=(0,0,-1,1,0)^T$, $R_3=(0,0,0,-1,1)^T$ and $R_4=(0,0,1,0,-1)^T$. The set of the conservation laws is

$$ \mathcal {M} =\operatorname {span} \{ (0,1,1,1,1)^T, (1, 0,1,1,1)^T \}. $$

Hence the system is conservative and $\mathcal {M}$-connected. Consider the effective chemical reaction network

$$ (2) \underset{K_{-1} }{\overset{K_1(f) }{\rightleftarrows }\ } (3) , \quad (3) \underset{K_{-2} }{\overset{K_2}{\rightleftarrows }\ } (4) \underset{K_{-3} }{\overset{K_3}{\rightleftarrows }\ } (5) \underset{K_{-4} }{\overset{K_4}{\rightleftarrows }\ } (3). $$

where $$K_1(f) = f(t) K_1 $$. Notice that $$\Gamma = ( \{ 2\}, \mathcal {R}_\Gamma )$$ where $$ \mathcal {R}_\Gamma = \{ (-1,1,0,0 )^T , (1,-1,0,0) \} $$ is a buffering structure indeed $$|\Omega _\Gamma | =1 $$, $$|\mathcal {R}_\Gamma | = 2 $$ and $$|\mathcal {C}_\Gamma |=1 $$. Hence $$\lambda (\Gamma )=0$$. However the signalling system does not have the property of adaptation since (1) and (3), (4), and (5) are connected by a conservation law. Hence the concentration of (3), (4), and (5) at steady state depends on the concentration (1) via a condition that depends on the fluxes $$J_R $$.

### A classical model of adaptation with fine tuned parameters

We start analysing the classical adaptation model proposed in Segel et al. (1986) by Segel, Goldbeter, Devreotes, and Knox. One of the goals of this section is to illustrate that, even if the kinetic system in Segel et al. (1986) is closed and satisfies the adaptation property, the results of that paper are consistent with Theorem 3.12. Indeed, the model in Segel et al. (1986) satisfies the adaptation property only for fine tuned parameters.

The model considered in Segel et al. (1986) aims at describing a signalling system that satisfies the adaptation property. This mechanism occurs in the cellular slime mold, *D. Discoideum*. These cells synthetize cAMP upon attachment of extracellular cAMP to the membrane receptors. The adaptation property of this mechanism is modelled in Segel et al. (1986) by assuming that the receptors can be at two different states. We present the chemical system studied in Segel et al. (1986). The substances of the network in Segel et al. (1986) are two types of receptors *D* and *R*, the ligands *L* and two types of complexes ligand receptor $X=[LR]$ and $Y=[LD]$. The signal in this case is the ligand. The set of the reactions taking place in the signalling system is

$$ (L) + (R) \underset{k_{-r}}{\overset{k_r}{\leftrightarrows }}(X), \quad (L) + (D) \underset{k_{-d}}{\overset{k_d}{\leftrightarrows }} (Y), \quad (R) \underset{k_{-1}}{\overset{k_1}{\leftrightarrows }} (D), \quad (X) \underset{k_{-2}}{\overset{k_2}{\leftrightarrows }} (Y). $$

We have two conserved quantities in this system, i.e. the number of receptors is conserved and the number of ligands is conserved

$$ R+D+X+Y=R_0\ \text { and }\ L+X+Y=L_0. $$

In particular this implies that the system is conservative. The reaction rates in Segel et al. (1986) are assumed to satisfy the detailed balance property (see equation (29) in their paper). As a consequence, the kinetic system is closed. Moreover, the conservation laws satisfy the $\mathcal {M}$-connectivity property. It is proven in Segel et al. (1986) that this signalling kinetic system satisfies the adaptation property, although only for fine tuned parameters. This result does not contradict Theorem 3.12 because the parameters must be fine tuned in order to have the property of adaptation.

By completeness, we briefly review the results obtained in the paper (Walz and Caplan 1987). In that paper the system of adaptation studied in Segel et al. (1986) is seen as an effective model obtained freezing some of the concentrations in a larger chemical network involving more substances. The results in Walz and Caplan (1987) have some analogies with Proposition 3.9, where we explain how to complete a signalling system that is non conservative to a conservative signalling system. The first extended model considered in Walz and Caplan (1987) consists in assuming that the reactions $(R) \leftrightarrows (D)$ and $(X) \leftrightarrows (Y)$ are obtained starting from reactions involving two additional substances, for instance the substances (*A*), (*B*) , that in the reduced system studied in Segel et al. (1986) are assumed to be constant in time. Then the completed reactions are of the form $(R) + (A) \leftrightarrows (D)+(B)$ and $(X) + (A) \leftrightarrows (Y) + (B)$. It is shown in Walz and Caplan (1987) that the detailed balance property of the kinetic system in Segel et al. (1986) follows from the detailed balance of this completed systems. The reason is that the cycles of the completed system and the kinetic system in Segel et al. (1986) are the same, i.e. we have that the space of the cycles for both the systems is

$$ \operatorname {span} \{ (1,-1,-1,1) \}. $$

This is consistent with Theorem 5.4 in Franco and Velázquez (2025a). Indeed this theorem states that if we consider a system with the detailed balance property and we freeze the concentration of certain substances in the network in such a way that the reduced network (involving only the non constant concentrations) and the original network have the same cycles, then the detailed balance of the reduced network follows directly from the detailed balance of the original network.

The second completion consist in assuming that the reactions $(R) \leftrightarrows (D)$ and $(X) \leftrightarrows (Y)$ are obtained as the reduction of reactions involving four additional substances, for instance the substances *A*, *B*, *W*, *Z*. Then the completed reactions are of the form $(R) + (A) \leftrightarrows (D)+(B)$ and $(X) + (W) \leftrightarrows (Y) + (Z)$. As mentioned in Walz and Caplan (1987), the detailed balance property of the kinetic system in Segel et al. (1986) can only be obtained assuming that the concentration of the additional substances (*A*), (*B*), (*W*), (*Z*) are chosen at equilibrium values. Indeed, it is easy to notice that the completed kinetic system does not have cycles while the kinetic system in Segel et al. (1986) has cycles. This is consistent with Proposition 5.6 in Franco and Velázquez (2025a) where it is proven that if the reduced system has more cycles than the completed systems then the only way to have that the detailed balance property of the reduced system holds in a robust manner is to choose the frozen concentrations at equilibrium values.

## Models exhibiting asymptotic adaptation

In this section we review some models of adaptation that can be found in the literature. In this paper we consider signalling systems in which the chemical reactions are bidirectional and where the chemical rates are of order one. Many examples of systems that exhibit the property of stable adaptation can be seen as limiting models of the signalling systems considered in this paper. These limiting models can be obtained assuming that certain parameters, or certain concentrations, tend either to zero or to infinity. In this section we explain in an heuristic manner how some classical models can be obtained starting from signalling systems with bidirectional reactions.

###  A linear version of the Barkai-Leibler model of robust adaptation for bacterial chemotaxis

In this section we briefly review a linear version of another classical model of robust adaptation, i.e. the Barkai-Leibler model of bacterial chemotaxis proposed in Barkai and Leibler (1997). In contrast with the model in Segel et al. (1986), this model satisfies the property of adaptation in a robust manner.

As we will see, the kinetic system studied in Barkai and Leibler (1997) is not a mass action kinetic system. The goal of this section is to give an idea on how this model can be derived starting from kinetic systems with mass action kinetics. To this end we need to make suitable assumptions on the speed of certain reactions taking place in the network.

The model proposed in Barkai and Leibler (1997) describes a particular mechanism of adaptation in the "run-tumble" swimming of E. Coli. These bacteria detect gradients of chemicals/signals (that can be an attractant or a repellent) in their vicinity. Their swimming is characterized by smooth runs, interrupted by events called tumbling, in which a new direction for the next run is chosen randomly. The bacteria are able to direct their motion towards attractants and away from repellents modifying the tumbling frequencies. The tumbling mechanism exhibits the adaption property, indeed the steady state tumbling frequency does not depend on the attractant/repellent concentration. However, the tumbling frequency can change if the bacteria perceives a change in time of the repellent/attractant concentration, i.e. if the bacteria perceives a gradient. This property allows bacteria to maintain their sensitivity to chemical gradients over a wide range of attractant or repellent concentrations.

A linear version of the model in Barkai and Leibler (1997) is given by the following system of ODEs

4.1 $$\begin{aligned} \frac{dX}{dt}&= Y - (1-c) X + f(t) \nonumber \\ \frac{dY}{dt}&= 1-c X, \end{aligned}$$

for a suitable $c>0$. Here *X* is the quantity of active receptors and *f* is the function describing the evolution of the signal, i.e. the evolution of the concentration of ligands (that could be attractants of repellents). Finally *Y* is the response regulator protein. The product of this system will be the quantity of active receptors *X*. Due to the form of the second equation in (4.1) at equilibrium the concentration of *X* does not depend on the asymptotic values of the signal *f*(*t*). It is also common in the literature of system biology to interpret the model (4.1) has a representation of the classical integral feedback controller (Alon 2019; Aoki et al. 2019; Frei et al. 2022).

The system of ODEs (4.1) does not correspond to a mass action kinetics, due to the loss term $$- c X $$ in the equation for *Y*. Indeed, mass action implies that the loss terms in the equation for a given substance *Y* must be proportional to the substance *Y* itself.

We briefly explain, without entering into the technical details, how this model can be obtained as a limit of mass action kinetic systems. We start considering the following bidirectional system

$$\begin{aligned}&X \overset{1}{ \underset{\varepsilon }{\leftrightarrows }} Z , \quad Y \overset{1}{\underset{\varepsilon }{\leftrightarrows }X}, \quad S \overset{1}{ \underset{\varepsilon }{\leftrightarrows }} X, \quad Z \overset{1}{\underset{\varepsilon }{\leftrightarrows }} Y \\&E+Y \underset{k_{-1}}{\overset{k_1}{\leftrightarrows }} [yE],\quad [yE]+ X\underset{k_{-2}}{\overset{k_2}{\leftrightarrows }}[xyE], \quad [xyE]\overset{\lambda }{ \underset{\varepsilon }{\leftrightarrows }} E+ P. \end{aligned}$$

Notice that this system satisfies the detailed balance property due to the absence of cycles. Moreover it is also conservative. Here *S* represents the signal, (*P*) the product of the enzymatic reactions, *E* an enzyme, [*yE*] is the complex formed by the enzyme and regulator protein, while [*xyE*] is the complex formed by the enzyme, the regulator protein and the active receptor.

In order to obtain a thermodynamically admissible system we freeze the concentration of *Z* to the value $N_Z$. The resulting reduced system that we obtain is then

$$\begin{aligned}&X \overset{1}{ \underset{\varepsilon N_Z}{\leftrightarrows }} \emptyset , \quad Y \overset{1}{\underset{\varepsilon }{\leftrightarrows }X}, \quad S \overset{1}{ \underset{\varepsilon }{\leftrightarrows }} X, \quad \emptyset \overset{1}{\underset{\varepsilon N_Z}{\leftrightarrows }} Y \\&E+Y \underset{k_{-1}}{\overset{k_1}{\leftrightarrows }} [yE],\quad [yE]+ X\underset{k_{-2}}{\overset{k_2}{\leftrightarrows }}[xyE], \quad [xyE]\overset{\lambda }{ \underset{\varepsilon }{\leftrightarrows }} E+ P . \end{aligned}$$

Taking the limit as $\varepsilon \rightarrow 0$ we obtain

4.2 $$\begin{aligned}&X \overset{1}{\rightarrow }\emptyset , \quad Y \overset{1}{\rightarrow }X, \quad S\overset{1}{\rightarrow }X, \quad \emptyset \overset{1}{\rightarrow }Y \end{aligned}$$

4.3 $$\begin{aligned}&E+Y \underset{k_{-1}}{\overset{k_1}{\leftrightarrows }} [yE],\quad [yE]+ X\underset{k_{-2}}{\overset{k_2}{\leftrightarrows }}[xyE], \quad [xyE]\overset{\lambda }{\rightarrow }E+ P . \end{aligned}$$

The system of ODEs corresponding to the enzymatic reactions in (4.3) are of the following form

$$\begin{aligned} \frac{dX}{dt}&= k_{-2} [xy E]-k_2 X [yE] \\ \frac{dY}{dt}&= -k_{1} E Y + k_{-1} [yE] \\ \frac{dE}{dt}&= -k_{1} E Y + k_{-1} [yE] + \lambda [xyE] \\ \frac{d[yE]}{dt}&= k_{1} E Y - k_{-1} [yE] +k_{-2} [xy E]-k_2 X [yE]\\ \frac{d[xyE]}{dt}&= - k_{-2} [xy E]+ k_2 X [yE] - \lambda [xyE]\\ \frac{dP }{dt}&= \lambda [xyE]. \end{aligned}$$

We then assume that the reaction $E+Y \underset{k_{-1}}{\overset{k_1}{\leftrightarrows }} [yE]$ and the reaction $[yE]+ X\underset{k_{-2}}{\overset{k_2}{\leftrightarrows }}[xyE]$ are very fast compared to the other reactions. This implies that the concentration of *Y*, *X* and of [*yE*] are essentially constant, as they reach steady state values very quickly. Therefore from the equations for the evolution of *Y*, *X* and of [*yE*] we deduce that

$$ [yE]= \alpha _{1} E Y, \ \text { and } \ [xy E]=\alpha _2 X [yE] = \alpha _1 \alpha _2 EY X $$

where $\alpha _1 = \frac{k_1}{k_{-1} }$ and $\alpha _2 = \frac{k_2}{k_{-2} }$. Notice that the system of ODEs above have a conserved quantity, i.e. we have that

$$ E+ [yE] + [xy E] =C_0. $$

From this we deduce that

$$ E+ \alpha _{1} E Y+ \alpha _1 \alpha _2 EY X =C_0, $$

hence $E=\frac{C_0}{1+ \alpha _1 Y+\alpha _1 \alpha _2 YX}$ and therefore

$$ [xy E]= \frac{ C_0 \alpha _1 \alpha _2 Y X}{1+ \alpha _1 Y+\alpha _1 \alpha _2 YX}. $$

Assume that $\alpha _2 \ll 1$ and that $\lambda \alpha _2 \approx 1$ (hence $\lambda \gg 1$) and that $X\approx 1$ and $Y\approx 1$. Then we deduce that

$$ \frac{dP }{dt}= \lambda [xyE] \approx (\lambda \alpha _2) \frac{ C_0 \alpha _1 Y X}{1+ \alpha _1 Y+\alpha _1 \alpha _2 YX}\approx \lambda \alpha _2 \frac{ C_0 Y X}{\frac{1}{\alpha _1}+ Y}. $$

If in addition we assume that $\alpha _1 \gg 1$ we obtain that

$$ \frac{dP }{dt}\approx \lambda \alpha _2 C_0 X. $$

Under the assumptions above the enzymatic reactions (4.3) can be reduced to the reaction

$$ Y \rightarrow P. $$

where the rate of the reaction depends on *X* and on the total number of enzymes, i.e. on $C_0$, more precisely the rate of the reaction is just $\lambda \alpha _2 C_0 X$. As a consequence, taking into account also of (4.2) we obtain the system of ODEs (4.1), where $c= \lambda \alpha _2 C_0$.

### An adaptation model of gene expression

In this section we study one of the models of adaptation considered in Ferrell (2016). This is a model of gene expression control. This model describes a system that produces of a transcription factor (which is the substance (4) in our notation) and that exhibits the adaptation property due to a mechanism of negative feedback. In this model the production of (4) is induced by another transcription factor (substance (5)) and by the signal (substance 1). The product inhibits the its own proliferation by binding to the transcription factor (4) and producing a complex (substance (6)) that eventually is degraded. This negative feedback yields the adaptation property.

The model that we are going to discuss, as well as all the models in Ferrell (2016), are one-directional networks. Instead in this paper we mostly deal with bidirectional kinetic systems. In this section we clarify the relation between bidirectional kinetic systems and one-directional kinetic systems. More precisely we want to explain heuristically that a one-directional kinetic system can be obtained as the limit of the bidirectional kinetic systems. Let us then consider the following set of chemical reactions, which is the kinetic system in Figure 5 in Ferrell (2016)

4.4 $$\begin{aligned} (1)+ (2) \rightarrow (3), \quad (2) \rightarrow \emptyset , \quad (3) \rightarrow (4), \quad (4)+(5) \rightarrow (6), \quad (5) \rightarrow \emptyset , \quad (5) \rightarrow (2). \end{aligned}$$

Notice that this system does not have cycles. The results in Gorban and Yablonsky (2011) imply that this system can be obtained starting from a bidirectional kinetic system in which detailed balance holds. The system above can be obtained starting from the following conservative system We start analysing the

$$\begin{aligned} (1)+ (2) \overset{K_1}{\underset{\varepsilon }{\leftrightarrows }} (3), \quad (2) \overset{K_2}{\underset{\varepsilon }{\leftrightarrows }} (7) , \quad (3) \overset{K_3}{\underset{\varepsilon }{\leftrightarrows }} (4), \quad (4)+(5) \overset{K_4}{\underset{\varepsilon }{\leftrightarrows }} (6), \quad (5) \overset{K_5}{\underset{\varepsilon }{\leftrightarrows }} (7) \quad (5) \overset{K_6}{\underset{\varepsilon }{\leftrightarrows }} (2) \end{aligned}$$

where $$K_i >0 $$ for every $$ i \in \{1,\dots , 6 \} $$. The first step in order to recover (4.4) as a thermodynamically admissible system is to freeze the concentration of (7) to obtain

$$\begin{aligned} (1)+ (2) \overset{K_1}{\underset{\varepsilon }{\leftrightarrows }} (3), \quad (2) \overset{K_2}{\underset{\varepsilon }{\leftrightarrows }} \emptyset , \quad (3) \overset{K_3}{\underset{\varepsilon }{\leftrightarrows }} (4), \quad (4)+(5) \overset{K_4}{\underset{\varepsilon }{\leftrightarrows }} (6), \quad (5) \overset{K_5}{\underset{\varepsilon }{\leftrightarrows }} \emptyset \quad (5) \overset{K_6}{\underset{\varepsilon }{\leftrightarrows }} (2). \end{aligned}$$

We then take the limit as $\varepsilon \rightarrow 0$ to recover (4.4).

## Conclusions and open problems

In this paper we prove that the property of robust adaptation can be achieved in signalling systems that satisfy the property of detailed balance. We identify two mechanisms yielding adaptation in systems with detailed balance. On one hand, we have obtained a large class of chemical networks for which the adaptation property holds in a robust manner. These systems are non-conservative, i.e. they model a system that exchanges chemicals with the surroundings. On the other hand, we also prove that closed signalling systems (i.e. conservative signalling systems with detailed balance) satisfy the property of robust adaptation only if the conservation laws satisfy a factorization assumption. This is due to the fact that one of the features that play an important role in the property of robust adaptation are the structural properties of the conservation laws in the model.

One of the main questions analysed in this paper is to understand the properties of systems that can be modeled using thermodynamically admissible systems of the form (1.3). Our definition of thermodynamically admissible systems assumes that the concentration of some substances in the system are frozen at constant values. Therefore, there are external fluxes of these substances into the system that keep these concentrations constant. This gives a particular way of exchanging mass with the surroundings. It would be interesting to study other mechanisms providing fluxes between the system and the environment.

In this paper we focus on signals that approach to a constant value as time tends to infinity. It would be possible to consider different situations. For instance it would be possible to consider periodic signals. It is an interesting question to understand which type of response we would have in a signalling system that satisfies the property of adaptation, as defined in this paper when the signal is periodic. One could imagine different situations. A relevant question in this case would be to ascertain if the response of the signallig system is periodic and if its average is independent on the average of the signal. We could also consider periodic signals, sinusoidal or squared signals, with a period that is much larger than the stabilization time of the signalling system or with a period which is much shorter than the stabilization time.

## Data Availability

Data sharing not applicable to this article as no datasets were generated or analysed during the current study.
